# Glycoproteomics identifies HOMER3 as a potentially targetable biomarker triggered by hypoxia and glucose deprivation in bladder cancer

**DOI:** 10.1186/s13046-021-01988-6

**Published:** 2021-06-09

**Authors:** Andreia Peixoto, Dylan Ferreira, Rita Azevedo, Rui Freitas, Elisabete Fernandes, Marta Relvas-Santos, Cristiana Gaiteiro, Janine Soares, Sofia Cotton, Beatriz Teixeira, Paula Paulo, Luís Lima, Carlos Palmeira, Gabriela Martins, Maria José Oliveira, André M. N. Silva, Lúcio Lara Santos, José Alexandre Ferreira

**Affiliations:** 1 Experimental Pathology and Therapeutics Group, Research Center (CI-IPOP), Portuguese Institute of Oncology, 4200-072 Porto, Portugal; 2 Institute of Biomedical Sciences Abel Salazar (ICBAS), University of Porto, 4050-313 Porto, Portugal; 3 Institute for Research and Innovation in Health (i3S), University of Porto, 4200-135 Porto, Portugal; 4 Institute for Biomedical Engineering (INEB), University of Porto, 4200-135 Porto, Portugal; 5 REQUIMTE-LAQV, Department of Chemistry and Biochemistry, Faculty of Sciences of the University of Porto, 4169-007 Porto, Portugal; 6 Cancer Genetics Group, Research Center (CI-IPOP), Portuguese Oncology Institute of Porto (IPO Porto), 4200-072 Porto, Portugal; 7 Immunology Department, Portuguese Institute of Oncology of Porto, 4200-072 Porto, Portugal; 8 Health School of University Fernando Pessoa, 4249-004 Porto, Portugal; 9 Department of Surgical Oncology, Portuguese Institute of Oncology, 4200-072 Porto, Portugal; 10 Porto Comprehensive Cancer Center (P.ccc), 4200-072 Porto, Portugal

**Keywords:** Glycomics, Glycoproteomics, Bladder cancer, Cancer microenvironment, Targetable biomarkers, Precision oncology

## Abstract

**Background:**

Muscle invasive bladder cancer (MIBC) remains amongst the deadliest genitourinary malignancies due to treatment failure and extensive molecular heterogeneity, delaying effective targeted therapeutics. Hypoxia and nutrient deprivation, oversialylation and *O*-glycans shortening are salient features of aggressive tumours, creating cell surface glycoproteome fingerprints with theranostics potential.

**Methods:**

A glycomics guided glycoproteomics workflow was employed to identify potentially targetable biomarkers using invasive bladder cancer cell models. The 5637 and T24 cells *O*-glycome was characterized by mass spectrometry (MS), and the obtained information was used to guide glycoproteomics experiments, combining sialidase, lectin affinity and bottom-up protein identification by nanoLC-ESI-MS/MS. Data was curated by a bioinformatics approach developed in-house, sorting clinically relevant molecular signatures based on Human Protein Atlas insights. Top-ranked targets and glycoforms were validated in cell models, bladder tumours and metastases by MS and immunoassays. Cells grown under hypoxia and glucose deprivation disclosed the contribution of tumour microenvironment to the expression of relevant biomarkers. Cancer-specificity was validated in healthy tissues by immunohistochemistry and MS in 20 types of tissues/cells of different individuals.

**Results:**

Sialylated T (ST) antigens were found to be the most abundant glycans in cell lines and over 900 glycoproteins were identified potentially carrying these glycans. HOMER3, typically a cytosolic protein, emerged as a top-ranked targetable glycoprotein at the cell surface carrying short-chain *O*-glycans. Plasma membrane HOMER3 was observed in more aggressive primary tumours and distant metastases, being an independent predictor of worst prognosis. This phenotype was triggered by nutrient deprivation and concomitant to increased cellular invasion. T24 *HOMER3* knockdown significantly decreased proliferation and, to some extent, invasion in normoxia and hypoxia; whereas *HOMER3* knock-in increased its membrane expression, which was more pronounced under glucose deprivation. HOMER3 overexpression was associated with increased cell proliferation in normoxia and potentiated invasion under hypoxia. Finally, the mapping of HOMER3-glycosites by EThcD-MS/MS in bladder tumours revealed potentially targetable domains not detected in healthy tissues.

**Conclusion:**

HOMER3-glycoforms allow the identification of patients’ subsets facing worst prognosis, holding potential to address more aggressive hypoxic cells with limited off-target effects. The molecular rationale for identifying novel bladder cancer molecular targets has been established.

**Graphical abstract:**

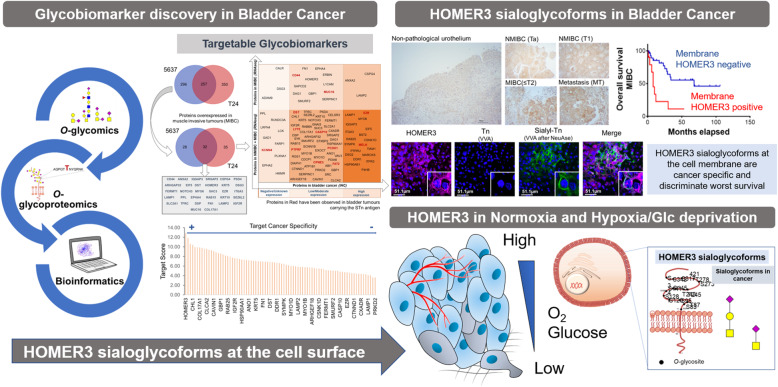

**Supplementary Information:**

The online version contains supplementary material available at 10.1186/s13046-021-01988-6.

## Background

Bladder cancer (BC) is one of the deadliest malignancies of the genitourinary system, especially in cases showing high cell dedifferentiation and muscle invasion [1]. Despite recent improvements in patient management provided by the introduction of immune checkpoint inhibitors, it mostly remains an orphan disease regarding novel effective targeted therapeutics [2]. Multi-target approaches constitute promising therapeutic strategies [3], reinforcing the quest for cancer biomarkers capable of maximizing therapeutic efficiency while reducing off-target effects.

The cell membrane proteome constitutes an important source of potentially targetable biomarkers, easily accessible to antibodies and other ligands [4]. The membrane proteome of cancer cells reflects their genetic, epigenetic, and transcriptomic instability, while actively contributing to all cancer hallmarks and disease progression [5]. The main events driving glycoproteome remodeling include changes in the protein abundance, primary structure, splicing mechanisms [6, 7] and, particularly, the nature, density and distribution of glycan chains, the main post-translation modification found at the cell surface [8, 9]. These changes may also translate in the formation of tumour-associated neoantigens, holding great potential for targeted therapeutics, including immunotherapy [10]. Therefore, targeting specific glycans may constitute an important starting point towards the identification of clinically relevant biomarkers and molecular targets.

Over four decades of research have disclosed a plethora of alterations in glycosylation pathways that significantly alter the membrane glycoproteome and consequently shape biomarker discovery [11]. The most striking stems from a drastic stop in protein *O*-glycosylation (occurring in Serine and/or Threonine residues) extension through sialylation, leading to the biosynthesis of sialylated short-chain *O*-GalNAc glycans, as sialyl-Tn (STn) and mono−/di-sialylated T antigens (herein termed ST antigens) [12–17]. The simplification of cancer cells *O*-glycome towards accumulation of these glycans has been suggested to be intimately linked to the downregulation of glycosyltransferases that extend glycan chains, as part of the cellular reprogramming necessary to support oxygen shortage (hypoxia) and nutrient deprivation derived from uncontrolled tumour growth and ineffective neovasculature [18–20]. Moreover, alterations in glycosylation under hypoxia are concomitant to the acquisition of mesenchymal phenotypes and increased cell invasion [18], suggesting that glycans may play an important role at this level that may be explored to target more aggressive cancer cells. Supporting these observations, glycoengineered BC cell models have demonstrated that STn overexpression alters protein functions in ways that favor cancer cell motility, invasion, metastasis, and immune escape, while being associated to unfavorable prognosis [17, 18, 21]. On the other hand, ST antigen overexpression mediated by *ST3GAL1* upregulation [13] appears to be part of the initial oncogenic transformation of the bladder, persisting in more advanced stages [22]. Despite its functional implications for cancer cells, glycans lack the necessary specificity to support the design of precise targeted therapeutics. However, zooming in on the glycoproteome for bispecificity may provide important fingerprints for distinct pathological contexts, holding true clinical potential. Showcasing this, we have described that MUC16-STn glycoforms (CA125 antigen) could define subsets of chemoresistant patients that were not evident based solely on the evaluation of the protein or the glycan [22]. Moreover, reduction in *O*-glycans length and changes in glycosites distribution may generate cancer-unique signatures in cancer-associated glycoproteins that may be explored for targeted therapeutics [10, 23]. These findings reinforce the importance of engaging in a comprehensive interrogation of the glycoproteome envisaging theranostics applications for BC.

## Material and methods

### Cell culture

The RT4, 5637, T24 and HT1197 bladder cancer cell lines reflecting different grades of the disease (I-IV) and major bladder carcinogenesis pathways were elected for this study (Table S1-Supporting Information). The cell lines were acquired from ATCC and cultured at 37 °C in a 5% CO_2_ humidified atmosphere and atmospheric oxygen (normoxia) with RPMI 1640 + GlutaMAX™ medium (Gibco™, Thermo Fisher Scientific; RT4, 5637 and T24) or Dulbecco’s High Glucose Modified Eagles Medium (HyClone™,GE Healthcare; HT1197) supplemented with 10% heat-inactivated FBS (Gibco, Thermo Fisher Scientific) and 1% penicillin-streptomycin (10,000 Units/mL penicillin; 10,000 mg/mL streptomycin; Gibco, Thermo Fisher Scientific). For experimental assays under glucose and/or oxygen shortage, cells were cultured in RPMI Medium 1640 (1x) without Glucose (Gibco™, Thermo Fisher Scientific) and maintained in a Galaxy 48R (New Brunswick) hypoxia chamber in a 99.9% N_2_ and 0.1% O_2_ humidified atmosphere.

### HOMER3 cell models

T24 cells were plated onto 24-well plates to be 70% confluent at the time of transfection. Human *HOMER3* (hHOMER3[NM_001145722.1]) knock-in (KI) was achieved by conventional mammalian gene expression vector transfection (VB191024-2277gen, VectorBuilder) using jetPRIME® transfection reagent (PolyPlus Transfection) according to the manufacturer’s instructions. In parallel, a mock system containing a 300 bp stuffer ORF was developed (VB191025-4255jjz, VectorBuilder). *HOMER3* knock-out (KO) was achieved using conventional plasmid DNA transfection of a mammalian gene expression vector (VB190513-1116eke, VectorBuilder) codifying *Streptococcus pyogenes* Cas9 and two gRNAs (gRNA1: TTTAGCCGCTCGCGCTCTGT, gRNA2: GCCAACACAGTCTACGGCCT) for maximum knock-out efficiency. Clonal selection of *HOMER3* KO was performed. Three KO clones were selected and clones with silent mutations provided phenotypic control cell lines. KI systems were optimized through puromycin selection of positively transfected cells. KI and KO models were validated by western blot and flow cytometry according to HOMER3 protein expression.

### Cell viability assay

Cell viability under hypoxia and glucose deprivation was determined using the propidium iodide (PI) exclusion test of cell viability. Briefly, cells cultured under normoxia and hypoxia plus glucose deprivation were detached using Accutase (Thermo Fisher Scientific) and stained with red-fluorescent PI nucleic acid binding dye (Thermo Fisher Scientific). Dead cells showing red fluorescence were quantified in a FC500 Beckman Coulter flow cytometer using the CXP Software. Results represent the standard deviation of three independent experiments.

### Proliferation assays

Cell proliferation was evaluated using the colorimetric Cell Proliferation ELISA, BrdU kit (Roche, Sigma-Aldrich) as previously described by Cotton, S. et al. [24]. All experiments were performed in triplicates.

### Invasion assays

Invasion assays were performed under normoxia and hypoxia plus glycose deprivation using Corning® BioCoat™ Matrigel® Invasion Chambers according to the vendors’ instructions and as described in Peixoto, A. et al. [18]. Invasion assays were normalized to cells proliferation index. Three independent assays were performed, and cells were seeded in duplicates. Results are presented as mean ± SD for each condition.

### Bladder cancer cells *O*-glycomics

Bladder cancer cellular models *O*-glycome was characterized through the Cellular O-glycome Reporter/Amplification method as previously described by Fernandes, E. et al. [23].

### Bladder cancer cells glycoproteomics

Plasma membrane proteins were extracted from whole cells by ultracentrifugation as previously described by Fernandes, E. et al. [23]. The final pellets corresponding to membrane proteins were resuspended in an appropriate volume of TBS/0.1% SDS. Plasma membrane enriched fractions were then digested with 10 U α-neuraminidase [*Clostridium perfringens* neuraminidase Type VI (Sigma-Aldrich)] and enriched for glycoproteins expressing the T antigen by agarose bound PNA lectin (Vector laboratories) chromatography. Protein extracts were then run on an SDS-PAGE gel and bands were excised from the gels, reduced with dithiothreitol (DTT; Sigma-Aldrich) and alkylated with iodoacetamide (Sigma-Aldrich). Glycopeptides were generated by trypsin digestion and analyzed by nanoLC-ESI-MS/MS in a nanoLC system (Dionex Ultimate 3000 RSLCnano) coupled online to an LTQ-Orbitrap XL mass spectrometer (Thermo Fisher Scientific) equipped with a nano-electrospray ion source (Thermo Fisher Scientific, EASY-Spray source), according to the conditions previously described by Cotton, S. et al. [22]. Data was analyzed automatically using the SequestHT search engine with the Percolator algorithm for validation of protein identifications (Proteome Discoverer 1.4, Thermo Fisher Scientific), using the conditions previously described [23, 25] Three variable modifications were considered, oxidation of methionine (+ 15.9 Da), carbamidomethylcysteine (+ 57 Da) and modification of serine and threonine with HexNAc (+ 203.1 Da; Tn antigen) and HexNAc-Hex (+ 365.1 Da; T antigen). Search engine retrieved identifications were curated using an in silico approach previously described [23, 25] and the resulting protein list was subsequently inspected for HexNAc and/or HexNAc-Hex *O*-glycosites by manual spectral annotation backed by GlycoPAT, a comprehensive open-source platform for MS-based glycoproteomics data analysis [26]. The final list of annotations included high confidence as well as medium/low confidence annotated glycoproteins exhibiting two or more glycopeptides showing HexNAc and/or HexNAc-Hex modifications. The same protocol was applied for identification of HOMER3 immunoprecitates after SDS-PAGE separation.

### Bioinformatics-assisted glycobiomarker discovery

The subset of plasma membrane proteins expressed by the cell lines was characterized according to their molecular and biological functions using the Search Tool for the Retrieval of Interacting Genes/Proteins (STRING) version 10.5 (http://string-db.org/) [27]. In addition, identified glycoproteins were categorized using Oncomine™ (https://www.oncomine.org/) [28] according to their overexpression in bladder cancer (non-muscle invasive bladder cancer-NMIBC; and MIBC) compared to healthy tissues, determined by RNAseq technology. A *p* ≤ 0.05 as well as a 2-fold variation of expression were considered. Expression of these proteins in bladder cancer and healthy tissues, determined by immunohistochemistry, was also assessed using The Human Protein Atlas (HPA; https://www.proteinatlas.org/). Protein expression in healthy tissues was stratified as “high”, “moderate”, “low”, “negative/not detected” and “not available” according to the HPA [29]. Regarding bladder tumours, five groups were created according to the number of patients expressing a given protein. Namely, when 80–100% of patients expressed a given protein, that group was considered a “high” expression group. Similarly, when 40–79%, 1–39, and 0% of patients expressed that protein, the groups were considered as “moderate”, “low” and “negative” expression groups, respectively. Whenever a protein was not addressed in bladder tumours, it was attributed to “unknown” expression group. The HPA also allowed determining the expression of identified glycoproteins in healthy human organs (brain, endocrine system, immune system, muscle tissues, respiratory, digestive, urinary and reproductive systems, adipose and soft tissues, and skin). This information was comprehensively integrated by a scoring system (*target score*) that ranks the identified glycoproteins in relation to their potential for targeted therapeutics with minimal off-target effects, as recently described by us [23, 24].

### Immunoprecipitation and Western blot

A Pierce™ Direct IP Kit (Thermo Fisher Scientific) was used according to the manufacturer’s instructions to selectively immunoprecipitate HOMER3 from membrane protein lysates using a rabbit polyclonal anti-HOMER3 antibody (PA5–59383, Thermo Fisher Scientific). The isolated glycoproteins were then run on 4–20% precast SDS-PAGE gels (Bio-Rad), transferred into nitrocellulose membranes and screened for HOMER3 and ST antigens expressions by western blot using the above-mentioned anti-HOMER3 antibody and a biotinylated PNA lectin (Vector laboratories), respectively. ST antigens detection by PNA lectin was preceded by overnight α-neuraminidase from *Clostridium perfringens* (Sigma-Aldrich) in membrane digestion 0.1 U/mL at 37 °C.

### Flow cytometry

The T and ST antigens expression in bladder cancer cell lines was determined by flow cytometry using fluorescein labelled PNA lectin (Vector laboratories), which preferentially binds to the T antigen. Adherent cells were detached using the non-enzymatic cell dissociation reagent Versene (Gibco™, Thermo Fisher Scientific) and fixed in 2% paraformaldehyde (PFA). Fluorescein labelled PNA lectin at 5 μg/mL was used for T antigen detection after 1 h incubation in PBS 2% FBS at room temperature under agitation in obscurity. ST antigens detection was achieved following the same protocol after overnight incubation at 37 °C with an α-neuraminidase from *Clostridium perfringens* (Sigma-Aldrich) at 70 mU/10^6^ cells. Since the lectin conjugate is supplied essentially free of conjugated fluorochromes, cell autofluorescence was used as negative control in every experiment. For total HOMER3 detection 2% PFA fixed cells were permeabilized with 0.2% Triton-X in PBS 1x for 5 min prior to staining with HOMER3 rabbit polyclonal antibody (PA5–59383, Thermo Fisher Scientific) at 16 μg/mL for 1 h at 4 °C. After washing, cells were incubated with goat anti-rabbit IgG (H + L) cross-adsorbed secondary antibody Alexa Fluor 488 (1:300, A-11008, Thermo Fisher Scientific) for 20 min at room temperature prior to detection. For membrane HOMER3 detection, non-permeabilized and non-fixed live cells were stained as above-described following staining with PI (Thermo Fisher Scientific) for dead cell gate exclusion. Rabbit IgG isotype controls were used for threshold signal definition (LTI-02-6102, Thermo Fisher Scientific). At least three concordant experiments were performed for each cell line and condition.

### Immunocytochemistry

To assess cell membrane and total expression of HOMER3, immunocytochemistry assays were performed. HOMER3 expression was determined in methanol fixed cells using a rabbit polyclonal antibody (PA5–59383, Thermo Fisher Scientific), incubated 1:100 for 1 h at room temperature. A goat anti-rabbit IgG (H + L) cross-adsorbed secondary antibody Alexa Fluor 594 (1:1000, A-11012, Thermo Fisher Scientific) was then incubated for 30 min at room temperature prior to detection. For total HOMER3 detection cells were permeabilized with 0.2% Triton-X in PBS 1x for 5 min. Nuclei were stained using Vectashield mounting medium (Vector Laboratories) with DAPI. Membrane HOMER3 was also assessed according to the flow cytometry optimized protocol, using PI as cell death control and CellMask™ Orange plasma membrane stain (Invitrogen) to demark cells at 0.5x for 6 min at room temperature. For ST antigens detection, 4% PFA fixed cells were incubated with 5 μg/ml of fluorescein labeled PNA lectin (Vector Laboratories) for 1 h at room temperature after 4 h of α-neuraminidase from *Clostridium perfringens* (Sigma-Aldrich) digestion 0.1 U/mL at 37 °C. Immunofluorescence images were acquired using a Zeiss Axio Imager Z1 (Carl Zeiss) microscope through a Axiocam MR ver3.0 (Carl Zeiss) camera and using the Software Axiovision 4.8 (Carl Zeiss).

### RNAseq for HOMER3 variants identification

Total RNA was extracted from cell pellets using the Qiagen RNeasy Plus Mini kit. RNA samples were quantified using Qubit 2.0 Fluorometer (Thermo Fisher Scientific) and RNA integrity was checked with Agilent TapeStation (Agilent Technologies). RNA sequencing library preparations used the NEBNext Ultra RNA Library Prep Kit for Illumina following manufacturer’s recommendations. Briefly, mRNA was first enriched with Oligo (dT) beads. Enriched mRNAs were fragmented for 15 min at 94 °C. First strand and second strand cDNA were subsequently synthesized. cDNA fragments were end-repaired and adenylated at 3’ends, and universal adapters were ligated to cDNA fragments, followed by index addition and library enrichment with limited cycle PCR. The sequencing libraries were validated on the Agilent TapeStation (Agilent Technologies), and quantified using a Qubit 2.0 Fluorometer (Invitrogen) and qPCR (KAPA Biosystems). The sequencing libraries were clustered on one lane of a flow cell, loaded on a Illumina HiSeq 4000 instrument and sequenced using a 2 × 150 Paired End configuration. Image analysis and base calling were conducted using HiSeq Control Software. One mismatch was allowed for index sequence identification. Garbage reads were trimmed from raw data and BAM files were generated. Unique gene hit counts were calculated using feature Counts from the Subread package v.1.5.2. Only unique reads that fell within exon regions were counted. Since a strand specific library preparation was performed, the reads were strand-specifically counted. After extraction of gene hit counts, the gene hit counts table was used for downstream differential expression analysis. A SNP/INDEL analysis was performed using mpileup within the Samtools v.1.3.1 program followed by VarScan v.2.3.9. The parameters for variant calling were minimum frequency of the alternate allele 25%, *p*-value less than 0.05, minimum coverage of 10, minimum read count of 7. A gene fusion analysis was performed using STAR Fusion v.1.1.0. For novel transcript discovery, transcripts expressed in each sample were extracted from the mapped bam files using Stringtie. The resulting gtf file was compared to the reference annotation file and novel transcripts were identified. The transcripts with ‘j’ in the fourth column of the tracking file are the novel transcripts.

### Gene expression

HOMER3 gene expression was assessed by quantitative polymerase chain reaction (qPCR). Briefly, total RNA from cultured cells was isolated using TriPure isolation Reagent (Roche), according to the manufacturer’s instructions. RNA conversion and gene expression analysis were performed as previously described [30] using TaqMan™ Gene Expression Assays (Applied Biosystems™). The mRNA levels were normalized to the expressions of *B2M* (Beta-2-microglobulin) and HPRT (Hypoxanthine-guanine phosphoribosyltransferase), which were found to be the most stable reference genes under the studied conditions and for the two cell lines [30]. The relative mRNA levels were calculated using the formula 2^−ΔΔCt^. The efficiency of each probe was above 95% as determined by the manufacturer. All reactions were run in duplicates and experiments were performed in triplicates.

### Patient samples and healthy human tissues

This study was performed retrospectively in a series of 104 formalin-fixed paraffin-embedded (FFPE) bladder tumours obtained from archived paraffin blocks at the Portuguese Oncology Institute of Porto (IPO-Porto). Patients were admitted and treated at IPO-Porto between 2000 and 2015. The series was composed by 53 non-muscle invasive bladder tumours, from which approximately 30% were Ta low grade tumors and the remaining were T1 high grade tumours. Fifty-one muscle invasive bladder tumours representative of all disease stages (12 tumours T2, 25 tumours T3, and 14 tumours T4) and 10 metastases biopsies (5 lymph-node metastases and 5 distant metastases) were also included. The time of follow-up was on average 49 months (1–226 months). Overall survival (OS) was defined as the period between surgery and patients’ death by cancer or between surgery and the last clinical visit. Clinicopathological information was obtained from patient’s clinical records. Healthy urothelium, thyroid, liver, gallbladder, testis, lung, stomach, pancreas, small intestine, colon, and appendix tissue sections were also included in the comparative study.

### Bladder tumors Glycoproteomics

Five MIBC tumours showing high STn and ST expressions, as determined by immunohistochemistry, were selected for tumour glycoproteomics. Proteins were extracted from FFPE tumour tissues using the Qproteome FFPE tissue kit (QIAGEN) according to the manufacturer’s instructions. Protein extracts were de-sialylated and enriched for Tn or T antigen expressing glycoproteins using VVA or PNA lectin (Vector Laboratories) affinity chromatography, respectively. Resulting glycoproteins were reduced, alkylated, and digested as described by Cotton, S. et al. [22]. Peptides were then separated using an UltiMate 3000 RSLC nano LC system (Thermo Fisher Scientific) equipped with a trapping cartridge (precolumn C18 PepMap 100, 5 μm, 300 μm i.d. × 5 mm, 100 Å) and an analytical column (Waters nanoEase HSS C18 T3, 75 μm × 25 cm, 1.8 μm, 100 Å). Solvent A was 0.1% formic acid in LC-MS grade water and solvent B was 0.1% formic acid in LC-MS grade acetonitrile. After loading the peptides onto the trapping cartridge (30 μL/min of 0.05% TFA in LC-MS grade water for 3 min), elution was performed with a constant flow of 0.3 μL/min using a 60 min analysis time (with a 2–28% B elution, followed by an increase to 40% B, and re-equilibration to initial conditions). The LC system was directly coupled to an Orbitrap Fusion Lumos ETD mass spectrometer (Thermo Fisher Scientific) using a Nanospray-Flex™ ion source (Thermo Fisher Scientific) and a Pico-Tip Emitter 360 μm OD × 20 μm ID, 10 μm tip (New Objective). The mass spectrometer was operated in positive ion mode with a spray voltage of 2.2 kV and a capillary temperature of 275 °C. Full scan MS spectra with a m/z range of 350–1800 were acquired in profile mode using a resolution of 120,000, maximum fill time of 50 ms or a maximum of 3e6 ions. Peaks with charge 2 to 8 and a minimum intensity of 5e4 ions (data-dependent acquisition) were selected for MS/MS acquisition. Precursor selection was allowed for up to 120 ms or until 1e5 ions have been collected (automatic gain control, AGC target). Fragmentation was performed using electron transfer/higher-energy collision dissociation (EThcD) with a preference for the highest charged peaks. The quadrupole isolation window was set to 3 Da and charge dependent electron transfer dissociation (ETD) parameters with supplemental Activation (HCD, 35%) were selected. MS/MS spectra were analyzed in an Orbitrap with a resolution of 15,000 in profile mode. Peaks were fragmented for a cycle time of up to 3 s. SEQUEST was set to consider z and c type ions and to allow tolerances of 5 ppm for precursor ions and 0.1 Da for fragment ions. Protein annotation was performed as described for glycoproteomics analysis of cell lines.

### Immunohistochemistry

FFPE tissue sections were screened for STn and ST antigens using a previously described streptavidin/biotin peroxidase method [17] as well as for HOMER3 using the Bond Polymer Refine Detection kit (Leica Biosystems), according to the manufacturer’s instructions. ST antigens were detected using 0,1 μg/mL biotinylated PNA lectin (Vector Laboratories) after a 4 h incubation with 0.2 mU/mL α-neuraminidase from *Clostridium perfringens* (Sigma-Aldrich) at 37 °C. STn antigen detection was achieved using an Anti-tag-72 antibody [B72.3 + CC49] (ab199002, Abcam) 0.5 μg/mL overnight at 4 °C, while HOMER3 was evaluated using a rabbit polyclonal antibody (PA5–59383, Thermo Fisher Scientific), 1:100 overnight at 4 °C. Immuno-reactive sections were blindly assessed using light microscopy by two independent observers and validated by an experienced pathologist. Briefly, ST antigen expression was scored according to a semi-quantitative approach based on the intensity and extension of the staining. The extension of staining was rated in cut-offs of 10%, and staining intensity was rated as follows: negative-0, weak-1, moderate-2, and strong-3 points. The tumours were then classified based on the multiplication of extension evaluation and intensity. Non-consensual readings were re-analyzed using a double-headed microscope and consensus was reached. For HOMER3 and STn antigens, classification was based on cellular location (cytoplasm or plasma membrane) and positivity was considered whenever the antigen was present.

### Double staining immunofluorescence microscopy

Glycans and HOMER3 were evaluated in the same tumor sections by double staining immunofluorescence microscopy. Briefly, FFPE bladder tumor sections were deparaffinized and rehydrated, followed by heat-induced epitope retrieval with citrate buffer pH 3.0. Unspecific background was blocked with Protein Block (Leica Biosystems). T antigen was stained using PNA-FITC lectin (Vector Laboratories) and Tn antigen was screened using VVA-FITC lectin (Vector Laboratories) 1:1000 for 1 h at room temperature. Their sialylated counterparts were stained using the same lectins after a 4 h incubation with 0.2 mU/mL α-neuraminidase from *Clostridium perfringens* (Sigma-Aldrich) at 37 °C. HOMER3 was evaluated using a rabbit polyclonal antibody (PA5–59383, Thermo Fisher Scientific), 1:100 overnight at 4 °C, following detection with an Alexa Fluor 594 goat anti-rabbit IgG (H + L) at the dilution of 1:100 for 30 min at room temperature. Nuclear counterstaining was obtained using a 4′,6-diamidino-2-phenylindole, dihydrochloride (DAPI; Thermo Fisher Scientific) solution for 10 min. Fluorescence images were acquired on a Leica DMI6000 FFW microscope (Leica Microsystems) using the Las X software (Leica Microsystems).

### Statistical analysis

Statistical analysis was performed using IBM Statistical Package for Social Sciences – SPSS for Windows (version 22.0; IBM, Armonk, NY, USA). T-tests and Two-way analysis of variance (ANOVA) Tukey tests were used to access the effect of hypoxia and glucose deprivation in HOMER3 expression in different models and subcellular locations. Chi-square (χ2) analysis was used to compare the categorical variables with a 5% level of significance. Fisher’s Exact Test was used for tables containing cells with less than 5 individuals. Kaplan–Meier survival curves were used to evaluate the correlation between overall survival (OS) and the evaluated biomarker. Comparison of estimates was done using log-rank tests. Further, multivariate Cox regression analysis was used to assess the independent prognostic marker value of HOMER3 on the OS and to adjust for potential confounders.

## Results

This work aimed at identifying glycobiomarkers of more aggressive forms of bladder cancer exploring a bioinformatics-assisted *O*-glycomics and *O*-glycoproteomics workflow, focusing on the glycosylation of serine and threonine residues. The main goal was to aid patient stratification and support the development of novel targeted therapeutics with limited off-target effects. Emphasis was also given to the role of hypoxia and glucose deprivation, two tumour microenvironmental features that impact on the glycoproteome in ways that favour invasion and poor prognosis [18, 31].

We started by conducting preliminary studies to elect cancer cell models showing more aggressive phenotypes translated by either increased proliferation or capacity to invade matrigel matrixes in vitro. Four widely studied bladder cancer cell models reflecting distinct histopathological natures were screened (RT4, 5637, T24, HT1197; Table S1-Supporting Information). According to Supporting Fig. S1, we elected for this study grade II 5637 and grade III T24 cell models, showing the highest invasion capacity. Moreover, T24 presented remarkably higher proliferation than the other cell models.

Envisaging glycobiomarkers discovery, BC cell lines were first submitted to *O*-glycomics characterization, generating key molecular information to guide glycoprotein identification. The identified glycoproteins were comprehensively integrated with available transcriptomics and proteomics data using bioinformatics tools. The objective was to pinpoint clinically relevant proteins for downstream validation, as highlighted by the scheme in Supporting Fig. S2. Most relevant glycobiomarkers were subsequently validated in patient samples and healthy human tissues envisaging the necessary molecular rationale to foster targeted therapeutics.

### Bladder cancer cell models *O*-glycome

**Fig. 1:**
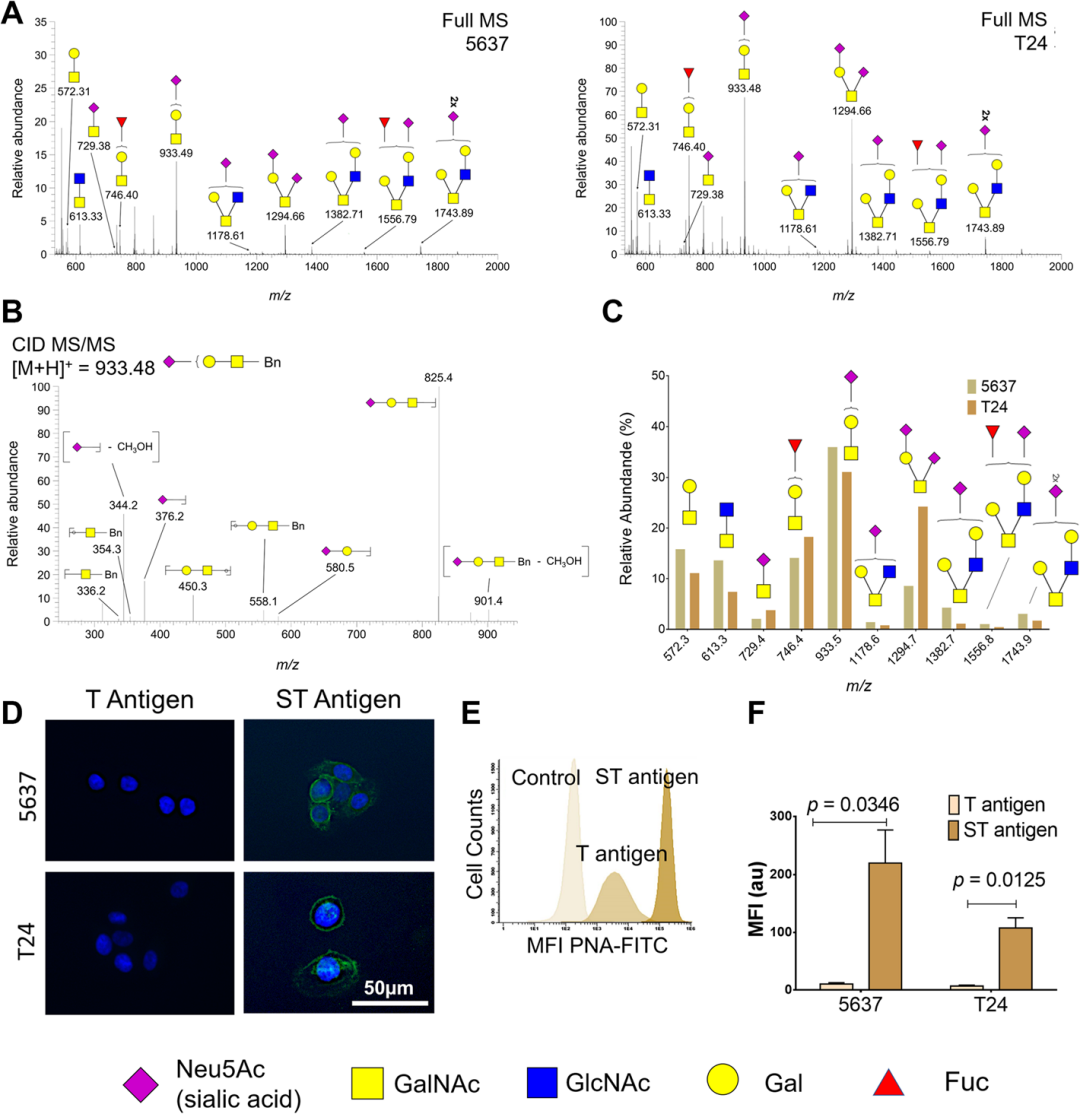
*O*-Glycome repertoire of 5637 and T24 bladder cancer cell lines showing a predominance of sialylated T antigens (sialyl-T and di-sialyl-T, herein generally termed ST antigens). **A**) Typical cellular *O*-Glycome Reporter/Amplification nanoLC-ESI-MS spectra for 5637 and T24 cells. Both mass spectra present mono and di-sialylated T antigens at *m/z* 933.5 and 1294.7 as dominant glycan species. Less abundant ions include T antigen (*m/z* 572.3), fucosylated T antigen (*m/z* 746.4) and sialylated and/or fucosylated core 2 glycans (*m/z* 1178.6, 1382.7, 1556.8, and 1743.9). Traces of the sialyl-Tn (STn) antigen could also be observed at *m/z* 729.4. **B**) MS/MS spectrum for the sialyl-T antigen at *m/z* 933.5. The MS/MS showed a major ion corresponding to the loss of the benzyl tag (Bn) and typical ions derived from glycosidic linkage cleavages (*m/z* 336.2, 354.3, 376.2, 450.3, 558.1, 580.5), corresponding to sugars diagnostic ions of sialyl-T structure. **C**) Relative quantification of major *O*-glycans contributing to the *O*-glycome for 5637 and T24 cells showing similar expression patterns. The graph shows that all cell lines present similar *O*-glycomes, emphasizing mono- and di-sialylated T antigens as the most abundant glycans. Notably, T24 cells appear enriched for di-sialylated T antigens whereas 5637 present a higher percentage of mono-sialylated species. **D**-**F**) Immunofluorescence microscopy and flow cytometry analysis of 5637 and T24 reveals very low levels of T antigens and an overexpression of sialylated species at the cell membrane. Panel D did not reveal T antigens but shows a significant accumulation of ST antigens at the cell surface. The histogram in panel E displays the expression of T antigen in bladder cancer cells before and after sialidase treatment for evaluation of ST antigens expression, highlighting the striking overexpression of ST antigens compared to its neutral form in these cell lines. Graph F compares T antigen levels after de-sialylation in the two cell lines, suggesting the higher expression in 5637 cells

*O*-glycomics analysis was performed using a glycan mimetic of the simplest form of *O*-glycosylation (benzyl-GalNAc, Bn-GalNAc). Briefly, Bn-GalNAc was first peracetylated to render it more hydrophobic and passively diffuse through the plasma membrane. The compound was then intracellularly deacetylated, glycosylated by the available cell glycosylation machinery and secreted back into the extracellular medium. After recovery by solid-phase extraction with a reversed-phase sorbent C18, Bn-*O*-glycans were permethylated to make it more hydrophobic, enabling downstream analysis by reverse-phase nanoLC-ESI-MS/MS [32]. A total of 10 major glycans structures were identified (Fig. 1A-C) for both 5637 and T24 cell models, which present similar *O*-glycomes (Fig. 1C). Notably, both cell lines expressed high levels of mono- and di-sialylated T antigens (herein generally termed ST antigens for simplification), also exhibiting neutral core 1 (*m/z* 572.3 and 746.4) and sialylated and/or fucosylated core 2 structures. Product ion spectrum of *m/z* 933.5 shows characteristic fragments resulting from glycosidic cleavage and confirms prior sialyl T identification (Fig. 1B). Trace amounts of the *m/z* 729.4 ion, corresponding to the sialyl-Tn (STn) antigen, a relevant short-chain *O*-glycan implicated in bladder cancer aggressiveness, were also observed in these cells. The low levels of STn in bladder cancer cell lines compared to bladder tumours reinforced the notion that this glycan expression is linked to microenvironmental cues that are not provided by conventional cell culture conditions [18]. Nevertheless, mono and/or di-sialylated T antigens were predominant glycan species in BC cell lines, in agreement with previous observations in bladder tumours [22]. The presence of sialylated T antigens at the cell surface was further confirmed by immunofluorescence microscopy (Fig. 1D) and flow cytometry using PNA lectin detection of the T antigen after sialidase digestion (Figs. 1E and F). Even though we did not use a cell surface marker, Fig. 1D showed clear membrane delimitation typical of ST antigens. According to Fig. 1D-F, both cell lines expressed residual amounts of the non-sialylated form of the T antigen as well as high levels of its sialylated form, in agreement with glycomics analysis. Interestingly, T antigen mono-sialylation T was more prominent in grade II 5637 than in grade III T24 cells, as suggested by previous reports [13]. On the other hand, di-sialylation was significantly enhanced in T24 cells. In summary, sialylated T antigens were the most abundant glycoforms presented by bladder cancer cell models, setting the necessary rationale for targeted glycoproteomics.

### Glycoproteomics and bioinformatics for targetable biomarkers

**Fig. 2:**
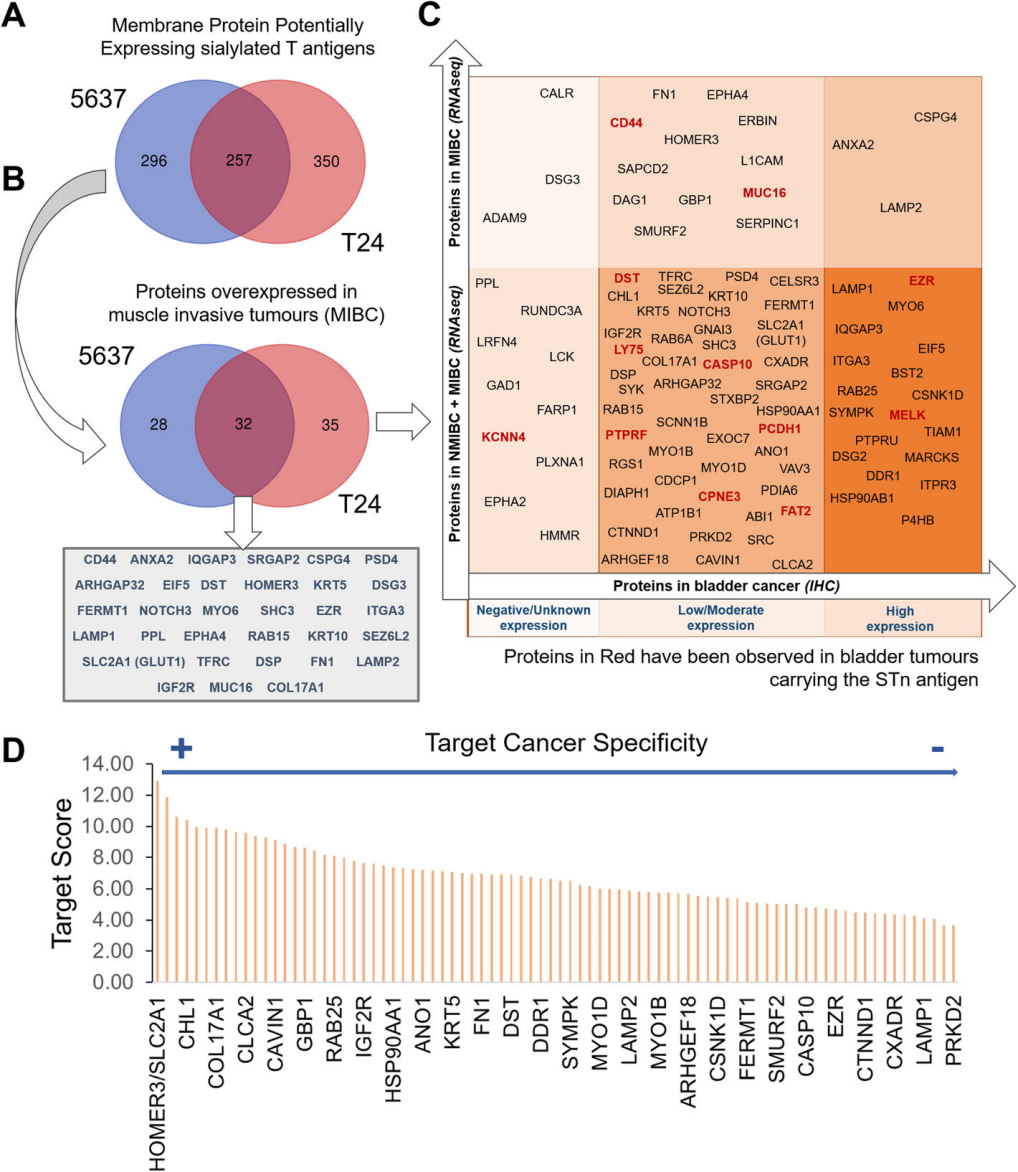
Identification of targetable glycoproteins in bladder cancer supported by bioinformatics analysis. **A**) Venn Diagram showing glycoproteins identified by targeted glycoproteomics focusing on sialylated T antigens in different cell models. A total of 903 glycoproteins were identified by mass spectrometry; however, only 28% were common to both cell lines, suggesting considerable distinct glycoproteomes. **B**) Venn diagram for glycoproteins overexpressed in MIBC according to the Oncomine (RNAseq) database. Glycoproteins were comprehensively matched with transcriptomic data in Oncomine to sort species potentially upregulated in MIBC in relation to non-pathological urothelium. Glycoproteins upregulated in both NMIBC and MIBC were also included for downstream analysis. 11% (95/903 assignments) were upregulated in MIBC, with the cell lines showing again very distinct and unique glycoproteomes. These glycoproteins were elected for specificity evaluation. **C**) Gene expression (Oncomine, RNASeq) vs protein levels (Human Protein Atlas; IHQ) for glycoproteins showing gene upregulation in MIBC. This diagram shows that 95 out of the 903 sorted glycoproteins were detected in MIBC, 81% of which were also present in NMIBC. The majority (63%) was present in low or moderate levels. A restricted group of 18 proteins, 3 of which highly expressed, were exclusively found in MIBC and may play a critical role in advanced disease. In addition, 13% (12/95) of the identified glycoproteins have been previously detected by targeted glycoproteomics for the STn antigen in bladder tumours showing resistance to chemotherapy (identified in red). **D**) Target Score for 29 glycoproteins showing some degree of expression in bladder tumours. Briefly, glycoproteins were ranked according to their potential for targeted therapeutics considering its plasma membrane expression in relation to other subcellular locations, absence from healthy urothelium and other human tissues, and overexpression in bladder cancer, while severely penalizing lymphoid system and gametes expression. The overall goal was to maximize tumour specificity while minimizing off-target effects. Maximum possible score was 15 (arbitrary units). GLUT1 (SLC2A1) and HOMER3 were top-ranked proteins scoring 13

Based on glycomics analysis, samples were first enriched for membrane proteins by differential ultracentrifugation of whole cell protein extracts, digested with sialidase and enriched for T antigen expressing glycoproteins by PNA affinity pulldowns. The glycoproteins were identified by conventional bottom-up proteomics after trypsin digestion and analysis by nanoLC-CID-MS/MS. Data was curated using a bioinformatics workflow for identification of glycoproteins with putative *O*-glycosylation sites. Overall, we have identified over 900 glycoproteins potentially expressing sialylated T antigens (607 for T24 cells and 553 for 5637 cells, 257 of which are common to both models; Fig. 2A); notably, *O*-glycopeptides could be detected in most of the identified glycoproteins (over 90%; Supplementary Tables S2–3), confirming glycoproteins annotations.

This dataset was then screened in silico for potentially targetable biomarkers associated with bladder cancer aggressiveness, combining transcriptomics and protein expression information from Oncomine and HPA databases, respectively. Oncomine showed that only 11% of the identified species (95/903; Fig. 2B) were upregulated in MIBC, 81% of which were also elevated in NMIBC (Fig. 2C). Notably, 32 of these glycoproteins could be detected in both cell lines (Fig. 2B), including biological and clinically relevant molecules in the context of disease such as CD44 and MUC16 [6, 22]. According to the HPA, more than 80% of upregulated glycoproteins had been detected in bladder tumours, 27% of which at very high levels (Fig. 2C). This database also showed that 9 glycoproteins were associated with worst prognosis (ANXA2, FN1, DSG3, CSPG4, IGF2R, GLUT1 (SLC2A1), DSP, MYO1D, CAVIN1). Moreover, a small subgroup of glycoproteins (12/95 marked red in Fig. 2C), including CD44 and MUC16, were previously found carrying cancer-associated glycan STn in MIBC [22, 33]. Interestingly, CD44-STn contributes to cancer tolerogenic immune responses and cell invasion, whereas MUC16-STn is expressed by subgroups of patients that do not respond to cisplatin-based therapeutics [21, 22]. Collectively, these findings support glycan targeting as a decisive tool for identification of clinically relevant glycoproteins. Data integration using bioinformatics narrowed down a list of over 900 entries to 95 glycoproteins potentially overexpressed in muscle invasive tumours, some already described as carriers of altered glycosylation in more aggressive tumours. In addition, several identified glycoproteins have also been associated with unfavorable prognosis, suggesting potential for targeted therapeutics.

Subsequently, glycoproteins were ranked according to their expression in bladder cancer and a wide array of human healthy tissues, in line with HPA insights (Supporting Fig. S3). The expression matrix in Fig. S3 served as basis for developing a ranking system (*target score*) to identify unforeseen cancer-specific molecular signatures, as previously described by us for glycobiomarker discovery in esophageal and gastric cancer [23, 24]. The scoring system significantly valued “low” or “null” expressions in healthy tissues, overexpression in bladder tumours and associations with poor prognosis. In addition, it favored proteins localized at the cell surface in cancer cells in opposition to other subcellular locations in healthy tissues, which is frequently observed in cancer [23, 34–36]. On the other hand, proteins present in lymphoid system intermediates and gametes were poorly scored, penalizing potential deleterious effects in immune and reproductive systems. As highlighted by Fig. 2D, GLUT1 and HOMER3 ranked first (scored 13/15), suggesting potential for targeted therapeutics. Interestingly, all the previously mentioned glycoproteins associated with worst prognosis scored lower (< 10/15), mostly due to their high expression in healthy tissues (Supporting Fig. S3). Namely, MUC16 scored 10/15 (not highlighted in the Target Score graph) and, more strikingly, CD44 (not highlighted in the Target Score graph), one of the most studied glycoproteins in bladder cancer, frequently suggested as a biomarker of cancer stem cells, scored 5/15 (Fig. 2D). As recently discussed by us, the exploitation of CD44 requires a comprehensive interrogation of its splicing variants mosaicism in health and disease for molecular signatures specifically associated with cancer [6]; however, to this date, it remains an unaddressed matter, significantly delaying guided therapeutics.

Focusing on top-ranked glycoproteins, GLUT1, encoded by the *SLC2A1* gene, is a pivotal membrane glucose transporter frequently overexpressed in more aggressive bladder tumours. GLUT1 overexpression has been associated to hypoxia, as part of the metabolic reprogramming accompanying transition from aerobic to anaerobic metabolism [37] and widely suggested as an independent prognostic factor [38]. Such observations support the potential of the *target score* system for pinpointing clinically relevant glycoproteins. On the other hand, Homer protein homolog 3 (HOMER3), encoded by the *HOMER3* gene, has been implicated in neuronal signaling, T-cell activation and trafficking of beta amyloid peptides [39–41]. Contrasting with GLUT1, HOMER3 is a poorly studied protein in cancer and, to our knowledge, is being suggested as glycosylated for the first time by this study (as highlighted by Supporting Figs. S4-S5). Moreover, bioinformatics analysis supported a restricted expression pattern in healthy tissues, suggesting potential for targeted therapeutics. As such, future studies should be focused on better understanding its functional and clinicopathological context in bladder cancer.

### HOMER3 in cell models

**Fig. 3:**
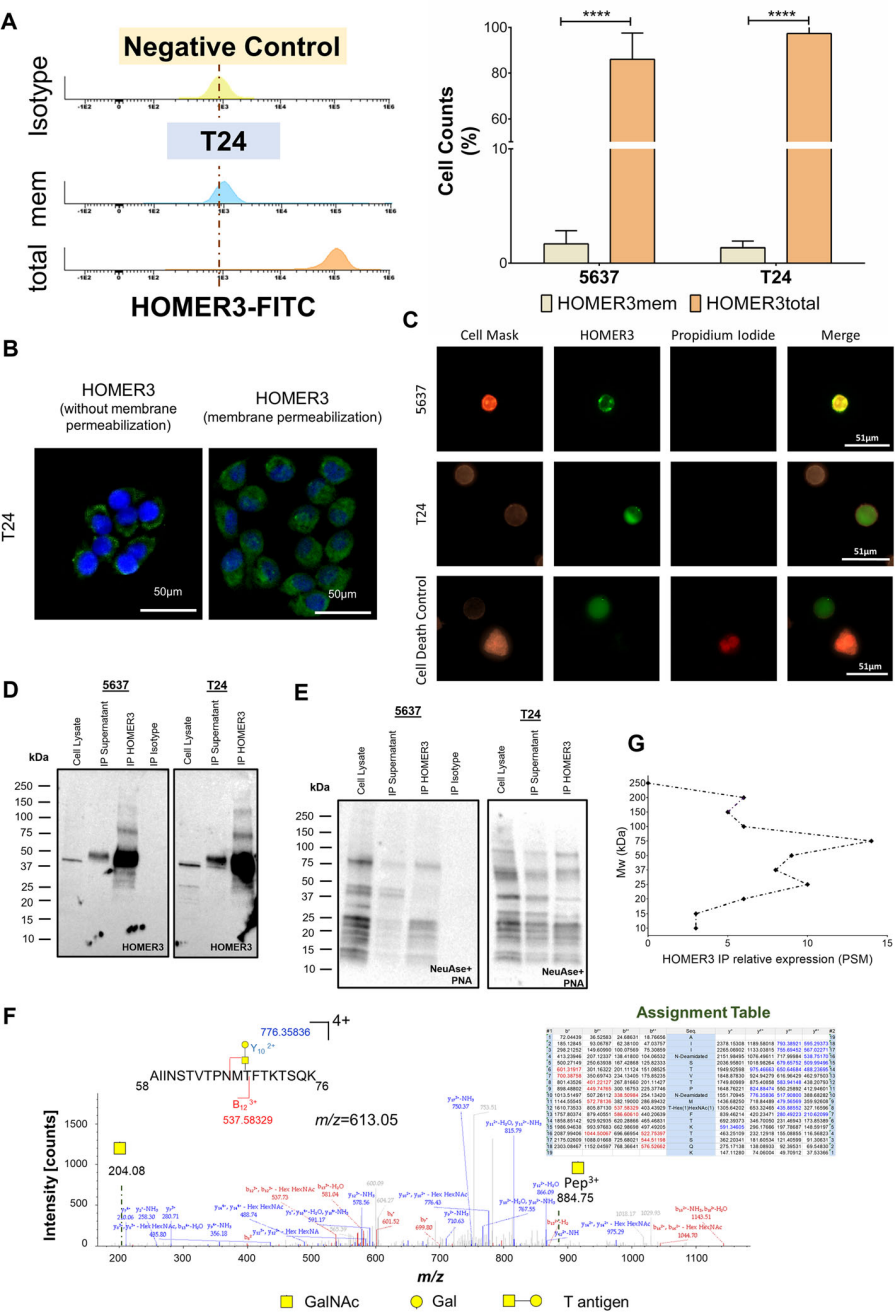
**A**) HOMER3 is expressed by the majority of 5637 and T24 cells (> 90%) showing mostly intracellular origin, whereas a small subpopulation also exhibits HOMER3 at the cell surface (3–5%). The left panel shows the expression of HOMER3 at the cell surface in a small subset of T24 cells (3–5%) and a massive expression of total HOMER3 (> 90% of the cells). The graph histogram at right highlights that 5637 and T24 cells presented the same HOMER3 phenotype. Experiments assessing HOMER3 at the cell surface were conducted in cells showing non-permeabilized membranes and without incorporation of propidium iodide. The analysis of permeabilized cells showed HOMER3 total levels. Results are the average of three independent replicates **** *p* < 0.0001 (student t-test). **B**) Immunofluorescence microscopy for HOMER3 (green) in non-permeabilized and permeabilized cells highlighting HOMER3 at the cell membrane (left panel). **C**) Immunofluorescence microscopy for HOMER3 (green) at the cell surface of non-permeabilized cells, as confirmed by a uniform expression of CellMask (orange) and the absence of signal for propidium iodine (red). Propidium iodine staining was used as cell death control for exclusion of cells with compromised plasma membranes (lower panel). In addition to PI, dead T24 cells also stained more intensely for cell mask than live cells due to dye diffusion into the intracellular space. **D**) Expression of HOMER3 in whole cell extracts, HOMER3 immunoprecipitates (IP) and corresponding supernatants. The blots clearly showed HOMER3 main proteoform at approximately 40 kDa, corresponding to its canonical form. HOMER3 IPs revealed additional bands at 75 and 100 kDa, potentially corresponding to glycoforms, and faint bands bellow 37 kDa. Both cell lines presented similar blot profiles. **E**) PNA lectin blots after in situ neuraminidase digestion (NeuAse) for whole cell extracts, HOMER3 immunoprecipitates (IP) and corresponding supernatants. Whole cell extracts show a wide number of bands spanning all molecular weights, consistent with the presence of multiple glycoproteins. Contrastingly, the IP showed bands at 75 and 100 kDa, reinforcing the existence of glycosylation. Moreover, no bands could be observed in the non-glycosylated HOMER3 canonical proteoform. Notably, several signals bellow 37 kDa were observed, suggesting low molecular weight HOMER3 glycoproteoforms. Again, both cell lines presented similar blot profiles. **F**) MS/MS spectra for a HOMER3 glycopeptide isolated at 75 kDa highlighting main assignments, peptide fragmentations as well as typical glycans oxonium ions at *m/z* 204.08 (GalNAc). **G**) Estimation of HOMER3 in IP bands by MS for T24 cells showed a wide number of proteoforms of distinct molecular weights (10–200 kDa), with higher abundance between 37 and 75 kDa. Bands were excised from gels, reduced, alkylated, subjected to proteolytic digestion, and analyzed by nanoLC-MS/MS. The presence of HOMER3 in each lane was estimated based on peptide-spectrum match (PSM), i.e. the total number of identified peptide spectra matched for the protein

We started by assessing membrane and total HOMER3 expressions in 5637 and T24 cell lines by flow cytometry, before and after permeabilization of the cell membrane. According to Fig. 3A, HOMER3 was present in significant amounts in both models (> 90% of the cells). However, the analysis of permeabilized compared to non-permeabilized cells in the presence of propidium iodide demonstrated that HOMER3 was mostly of intracellular origin (Fig. 3A). Contrastingly, only a small subpopulation of cells (3–5%) presented HOMER3 at the cell surface, revealing a restricted expression pattern (Fig. 3A). Notably, the expression of HOMER3 at the cell membrane in non-permeabilized cells was extinguished after mild trypsin digestion, reinforcing its localization at the cell surface (data not shown). Such observations were further reinforced by fluorescence microscopy showing a clear HOMER3 signal at the plasma membrane in non-permeabilized and live cells (Fig. 3B-C).

HOMER3 was then immunoprecipitated from plasma membrane proteins enriched extracts and blotted for both HOMER3 and sialylated T antigens by PNA lectin after neuraminidase digestion in situ. Whole cell extracts presented a main band at 40 kDa consistent with HOMER3 canonical form, which was also the most intense band in HOMER3 immunoprecipitates. However, IPs also showed less intense bands at 75 and above 100 kDa, suggesting an increase in molecular weight due to glycosylation (Fig. 3D). According to bioinformatics predictions with NetNGlyc and NetOGlyc, HOMER3 may putatively exhibit at least 18 *O*- and 2 *N*-glycosylation sites (Fig. S6). Such a high number of glycosites may significantly increase HOMER3 molecular weight, reinforcing these signals. The PNA blots confirmed the presence of glycosylation in higher molecular weight proteoforms (Fig. 3E), also supporting these observations. Moreover, PNA reactivity was not evident at approximately 40 kDa, corresponding to the most abundant canonical form of the protein. Notably, PNA blots also showed several intense bands bellow 37 kDa, which were not so evident when blotting for HOMER3 (Fig. 3E). and may be derived from less abundant glycosylated proteoforms. Although these signals could result from undesirable non-specific immunoprecipitation, we hypothesize that they may be derived from less abundant low molecular weight HOMER3 proteoforms that could be predicted from RNAseq data. To assess these hypotheses, HOMER3 IP bands were excised from the gel and analyzed by nanoLC-MS/MS. Still, we could only confirm by MS/MS analysis glycosylation at the 75 kDa and 100 kDa proteoform, as highlighted by the MS/MS spectra in Fig. 3F showing a wide number of peptide backbone fragmentations as well as typical glycans oxonium ions at *m/z* 204.08. However, as highlighted by the graph in Fig. 3G, HOMER3 could be detected at different molecular weights (spanning 10–200 kDa). According to exploratory RNAseq analysis, 5637 and T24 cells may express multiple HOMER3 isoforms, including several low molecular weight variants (Fig. S7, Table S4), reinforcing this hypothesis. Future confirmation by glycoproteogenomics approaches is warranted to disclose the existence of other HOMER3 glycoforms.

In summary, the membrane HOMER3 phenotype can be found in minor subpopulations of both 5637 and T24 cells. Moreover, HOMER3 is present at the cell surface predominantly as high molecular weight proteoforms carrying short-chain *O*-glycans typical of membrane proteins.

### HOMER3 functional impact under hypoxia and glucose deprivation

Hypoxia and glucose deprivation, often experienced by cancer cells in poorly vascularized niches, lead to profound remodeling of the membrane proteome [42, 43], including the translocation of intracellular proteins to the cell surface [44]. Therefore, we hypothesized that hypoxia and glucose deprivation, could drive HOMER3 expression and subcellular localization. Interest was devoted to understanding the potential functional implications of these alterations for cancer progression.

**Fig. 4:**
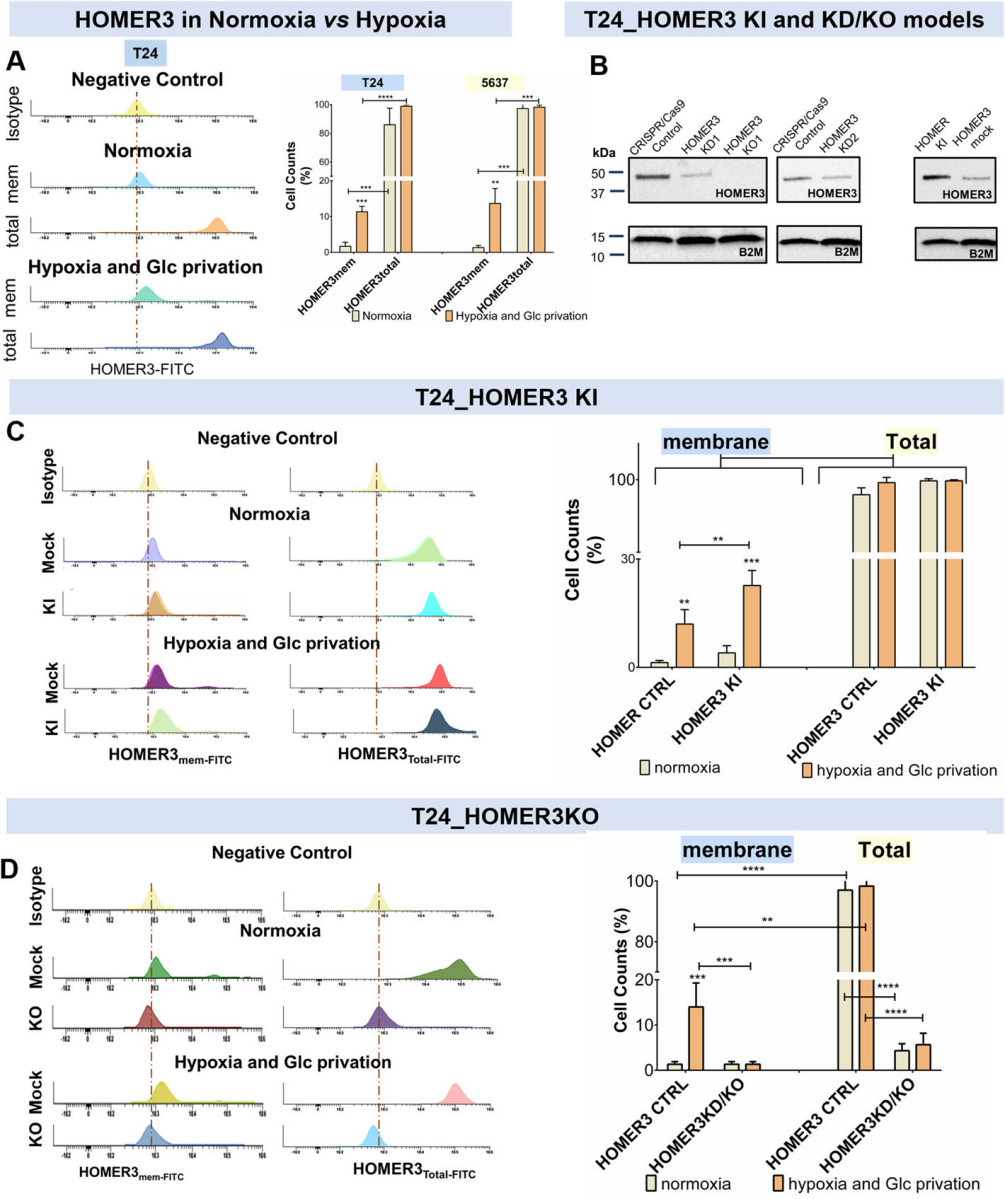
Hypoxia and glucose deprivation drive the accumulation of HOMER3 at the cell membrane. **A**) Hypoxia and Glucose deprivation significantly increased the number of T24 and 5637 cells expressing HOMER3 at the cell surface. T24 flow cytometry analysis (included in the left panel as a representative example) and graph A (right panel) shows that the vast majority of T24 and 5637 cancer cells express HOMER3, which remains unchanged upon tremendous reduction in oxygen levels and glucose deprivation. On the other hand, this microenvironmental feature promoted a massive increase of cells (approximately 5-fold) showing HOMER3 at the cell surface. **B**) Induction of HOMER3 upregulation and downregulation in T24 cells. Blots confirm the development of two HOMER3 knockdown (T24_HOMER3_KD1 and KD2; 30–50% decrease), one knockout model (T24_HOMER3_KO; total HOMER3 abrogation) and an HOMER3 knock-in (T24_HOMER3_KI; 60% increase compared with the control). **C**) T24 overexpression is mainly observed at the cell surface after exposure to hypoxia and glucose deprivation. The percentage of cells expressing HOMER3 increases significantly in relation to the controls in both normoxia and hypoxia plus glucose deprivation; however, this increase is more pronounced under microenvironmental pressure. **D**) HOMER3 downregulation or deletion leads to a massive decrease in the number of cells expressing the protein in normoxia, irrespectively of the sub-cellular localization, which is not compensated by exposure to hypoxia and glucose deprivation. The results are the average of at least three independent replicates. **p < 0.01; ***p < 0.001; **** *p* < 0.0001 (two-way ANOVA with interaction followed by Tukey post hoc)

We started by exposing bladder cancer cell lines to hypoxia and glucose deficiency, which led to a massive increase in lactate in the culture media for both 5637 and T24 cells (data not shown), in accordance with our previous reports [18, 45]. This demonstrated a metabolic reprogramming in response to the imposed microenvironmental challenge, as previously described by others [46–49]. Nevertheless, both cell lines tolerated well the experimental conditions, showing minimal cell death after 24 h (≈90% viability). Moreover, no significant changes were observed in *HOMER3* gene expression (data not shown) and total protein levels in both cell lines (Fig. 4A). Strikingly, the reduction in oxygen and lack of glucose induced a massive relocation of HOMER3 to the cell surface (3–5-fold increase in relation to normoxia; Fig. 4A) in the two models.

We then edited the genome of T24 cells through CRISPR-Cas9 technology to induce *HOMER3* knockdown (KD) or knock-out (KO). We also used conventional mammalian gene expression vector transfection for *HOMER3* knock-in (KI). Alterations in HOMER3 were later confirmed by western blot and flow cytometry (Figs. 4B, C and D). One KO and two KD clones were isolated, showing a decrease between 30 and 50% (T24HOMER3_KD1–2) and complete HOMER3 abrogation (T24HOMER3_KO; Fig. 4B). On the other hand, the T24HOMER3_KI model showed a 2-fold increase in HOMER3, which was more evident at the cell membrane (Fig. 4B). Notably, the accumulation of HOMER3 at the cell surface was significantly amplified under hypoxia and glucose deprivation (Fig. 4C), supporting the role played by the microenvironment in the translocation of proteins to the cell surface and, potentially, the extracellular space. In clear contrast, KD/KO models did not display the membrane phenotype neither in normoxia nor hypoxia (Fig. 4D). A massive reduction in total HOMER3 could also be observed for all conditions, in accordance with the western blots (Figs. 4B and D).

**Fig. 5:**
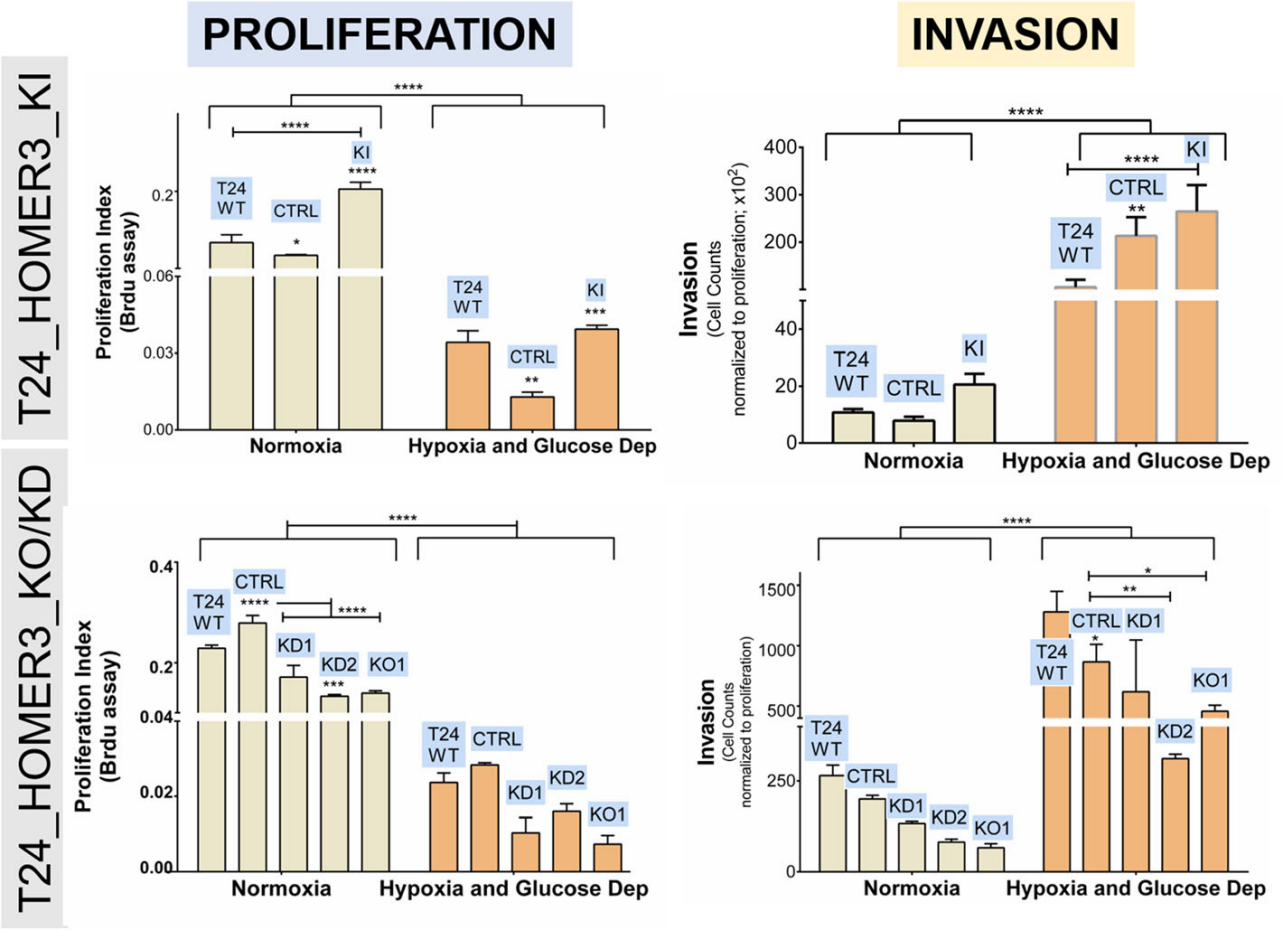
HOMER3 enhances T24 cells proliferation and invasion in normoxia and, particularly, under oxygen shortage and glucose deprivation. T24 wild type cells (T24WT), CRISPR-Cas9 control cells (CTRL), T24_HOMER_KI (with HOMER3 elevation) and KD1–2/KO1 (with HOMER3 downregulation/abrogation) models, all showed decreased proliferation and increased invasion in hypoxia plus glucose deprivation in comparison to normoxia. Notably, T24_HOMER3_KI models were significantly more proliferative and invasive in comparison to wild type and control cells in normoxia and, particularly, under hypoxia and nutrient shortage. The T24_ HOMER3_KD/KO showed the opposite behavior. The results are the average of at least three independent replicates. *p < 0.05; **p < 0.01; ***p < 0.001; **** *p* < 0.0001 (two-way ANOVA with interaction followed by Tukey post hoc)

Finally, we addressed the functional implications of different HOMER3 phenotypes in proliferation and cell invasion/motility. We started by observing a striking decrease in T24 cells proliferation after deprivation of oxygen and glucose (Fig. 5), in agreement with our previous observations [18, 45]. This was accompanied by an increase in cell invasion, which was tremendously amplified when normalizing the results to cell proliferation (Fig. 5). These findings suggested that deprivation of oxygen and glucose acted as a selective pressure towards the selection/establishment of more aggressive and potentialy quiescent cancer cells, as widely described by different authors for different cancer models [45, 50–52]. Similar experiments conducted using HOMER KI, showed that *HOMER3* upregulation induced proliferation in normoxia as well as in hypoxia in comparison to controls and wild type cells (Fig. 5). On the other hand, KO/KD models were significantly less proliferative in normoxia and a similar trend was observed for all clones under hypoxia, whose significance may be masked by the massive stop in cell proliferation induced by this microenvironmental condition. KI models showed a trend proliferation increase in normoxia and hypoxia plus glucose deprivation, contrasting with the observations fostered by HOMER3 KO/KD models, which were statistically significant under hypoxia and glucose deprivation. Collectively, we have demonstrated the pivotal role played by HOMER3 as promoter of cell proliferation and, to some extent, invasion under specific microenvironmental conditions. Overall, the functional role played by HOMER3 at the cell membrane appears to be dependent on microenvironmental cues. Namely, in normoxic conditions it decisively contributes to cell proliferation, whereas under low oxygen pressure and lack of glucose this becomes attenuated, reflecting the preponderant effect induced by the condition itself. Upon microenvironmental challenges, HOMER3 supported invasion, most likely endowing cells with escape mechanisms, suggesting bladder cancer cells adaptive responses. Therefore, our observations strongly suggest that HOMER3 translocation to the cell surface may be a key event driving cancer aggressiveness.

### HOMER3 expression in bladder tumours

**Fig. 6:**
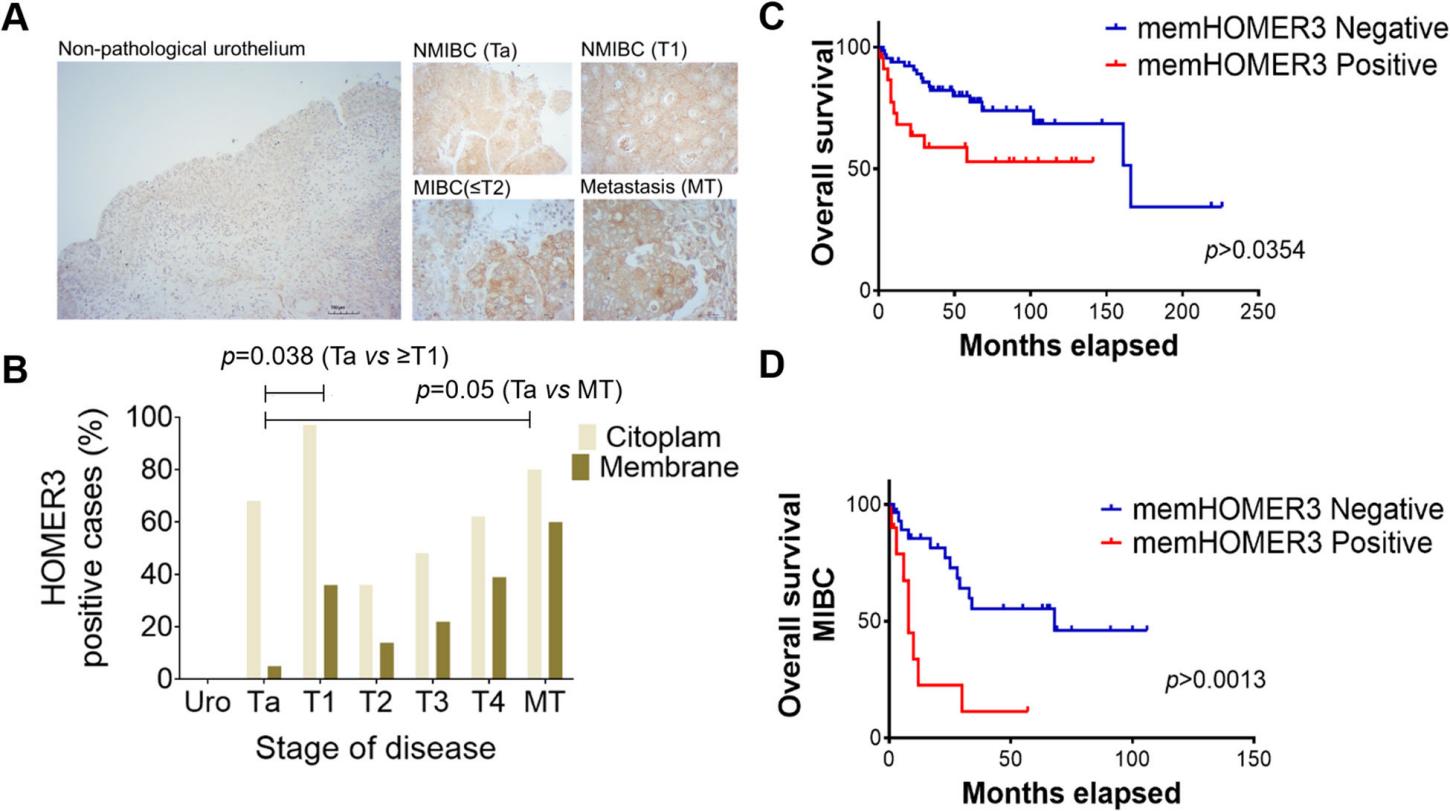
The HOMER3 membrane phenotype associates with bladder cancer aggressiveness and worst prognosis. **A**) HOMER3 is not expressed by the healthy urothelium but could be found at different bladder cancer stages (Ta-T4) and in metastases, with significant accumulation at the cell membrane for high-grade NMIBC and MIBC bladder cancer. HOMER3 was scored based on the number of positive cases and sub-cellular location (cytoplasm vs membrane and cytoplasm). HOMER3 was not expressed in healthy urothelium. On the other hand, the number of positive cases showing membrane staining increased with the severity of the lesions, being also present in most metastases. A significant association between HOMER3 at the cell membrane and high-grade tumours of stages T1 and higher could be observed (Ta vs ≥ T1: 5.3% vs 28.4%; *p* = 0.038; Chi-square). **B**) The overall survival of bladder cancer patients exhibiting HOMER3 at the plasma membrane (memHOMER3) was reduced compared to patients not showing this molecular feature. The survival curve in the upper panel highlights the association between the memHOMER3 and decreased OS (83 vs 148 months; log-rank *p* = 0.035). The lower panel shows that this is also true for the MIBC subgroup (15 vs 64 months; log-rank *p* = 0.001)

We then screened a broad number of tumours, metastases biopsies and histologically normal urothelium from healthy individuals for HOMER3. HOMER3 exhibited a focal expression pattern mostly restricted to basal cells in NMIBC and a less distinctive profile in MIBC (Fig. 6A). Given its low expression levels, HOMER3 was categorized as positive or negative without accounting for intensity and extension of the marked area. HOMER3 was predominantly found in the cytoplasm of cancer cells in most tumours (Supporting Fig. S8-left panel); however, a subgroup of approximately 25% of the patients with ≥ T1 tumours also exhibited membrane expression (Supporting Fig. S8-right panel). Interestingly, the membrane/cytoplasm ratio increased with the stage of the disease (0.07 in Ta; 0.38 in T1; 0.33 in T2; 0.46 in T3; and 0.63 in T4; Fig. 6B), suggesting that the plasma membrane anchoring of HOMER3 could be a molecular feature associated with tumour aggressiveness. Notably, we observed a higher percentage of T1 tumours showing HOMER3 at the cell membrane in comparison to higher stage lesions. Future studies should be conducted to confirm this association and the possibility of considering HOMER3 as a molecular marker of more aggressive non-muscle invasive disease. HOMER3 was also detected in 60% of the metastasis at the cell membrane, reflecting the percentage of positive primary tumours. Moreover, a clear association was observed between HOMER3 at the cell membrane and high-grade tumours of stages T1 and higher (Ta vs ≥ T1: 5.3% vs 28.4%; *p* = 0.038) as well as lymph node metastasis (Ta vs MT; *p* = 0.05; Fig. 6A). In contrast, this was not observed for the HOMER3 cytoplasmatic phenotype. In addition, HOMER3 was not detected in healthy urothelium, which supports its cancer-associated nature (Fig. 6A).

Facing these observations, we evaluated possible associations of HOMER3 with patient OS. Total HOMER3 levels were neither associated with the type of disease nor with overall survival. However, the subgroup of patients exhibiting membrane HOMER3 presented decreased OS (mean: 83 vs 148 months; log-rank *p* = 0.035; Fig. 6B), irrespectively of the type of disease. Notably, the expression of HOMER3 at the cell membrane in patients with MIBC, which generally present worst prognosis, was also associated with decreased OS in bladder cancer (mean: 15 vs 64 month; log-rank *p* = 0.001; Fig. 6B). Multivariate Cox regression adjusted to cases facing worst prognosis confirmed that the membrane HOMER3 phenotype was a significant independent biomarker of poor prognosis (Hazard Ratio 3.87; *p* < 0.002), which is consistent with the functional role exhibited by the glycoprotein.

### Short-chain sialylated *O*-glycans in bladder tumours

In parallel to HOMER3 detection, we have screened the same patient cohort and healthy urothelium with PNA lectin before and after sialidase treatment to assess T and sialylated T antigens, respectively. The T antigen was detected in approximately 5% of tumours, at very low levels, predominantly in non-muscle invasive lesions (Ta and T1) but not in the healthy urothelium (Supporting Fig. S9A). On the other hand, its sialylated form was observed in 70% of non-pathological urothelium sections (7/10) and 90% of the tumours (50/60), irrespectively of the histological nature (Supporting Fig. S9A). Fig. S9A also highlights that sialylated T antigens levels increased at initial stages of the disease (Ta) and decreased to basal levels when the tumours invaded the lamina propria/connective tissue (T1), muscle of the bladder wall (T2), fat layer and pelvic organs (≥ T3). Moreover, Ta tumours presented significantly higher extension and intensity of expression in comparison to more aggressive tumours. Despite this reduction in most aggressive cases, ST antigens were present in more than 20% of the tumour area, thus in agreement with the high ST content also found in cell models. In addition, we explored possible associations between ST and prognosis, which were not observed. Interestingly, the expression pattern of ST antigens significantly differed from the presented by the STn antigen (Fig. S9B), a shorter sialylated *O*-glycan implicated in an onset of bladder cancer hallmarks such as invasion and immune escape and extensively studied by us [12, 18, 21]. STn was absent from the healthy urothelium and significantly overexpressed in more aggressive forms of the disease, in clear contrast with ST antigen. Notably, all STn positive tumours also abundantly expressed sialylated T antigens. Such findings are in accordance with our previous reports [22]. It also reinforces the hypothesis that ST overexpression instead of more elongated structures may be part of the initial oncogenic events of the bladder. More profound *O*-glycans shortening translated by STn expression may be a surrogate of disease progression supported by profound alterations in glycosylation. It also provides key glycomics rationale for targeting HOMER3 at the cell-surface.

### HOMER3 glycoproteomics in bladder tumours

**Fig. 7:**
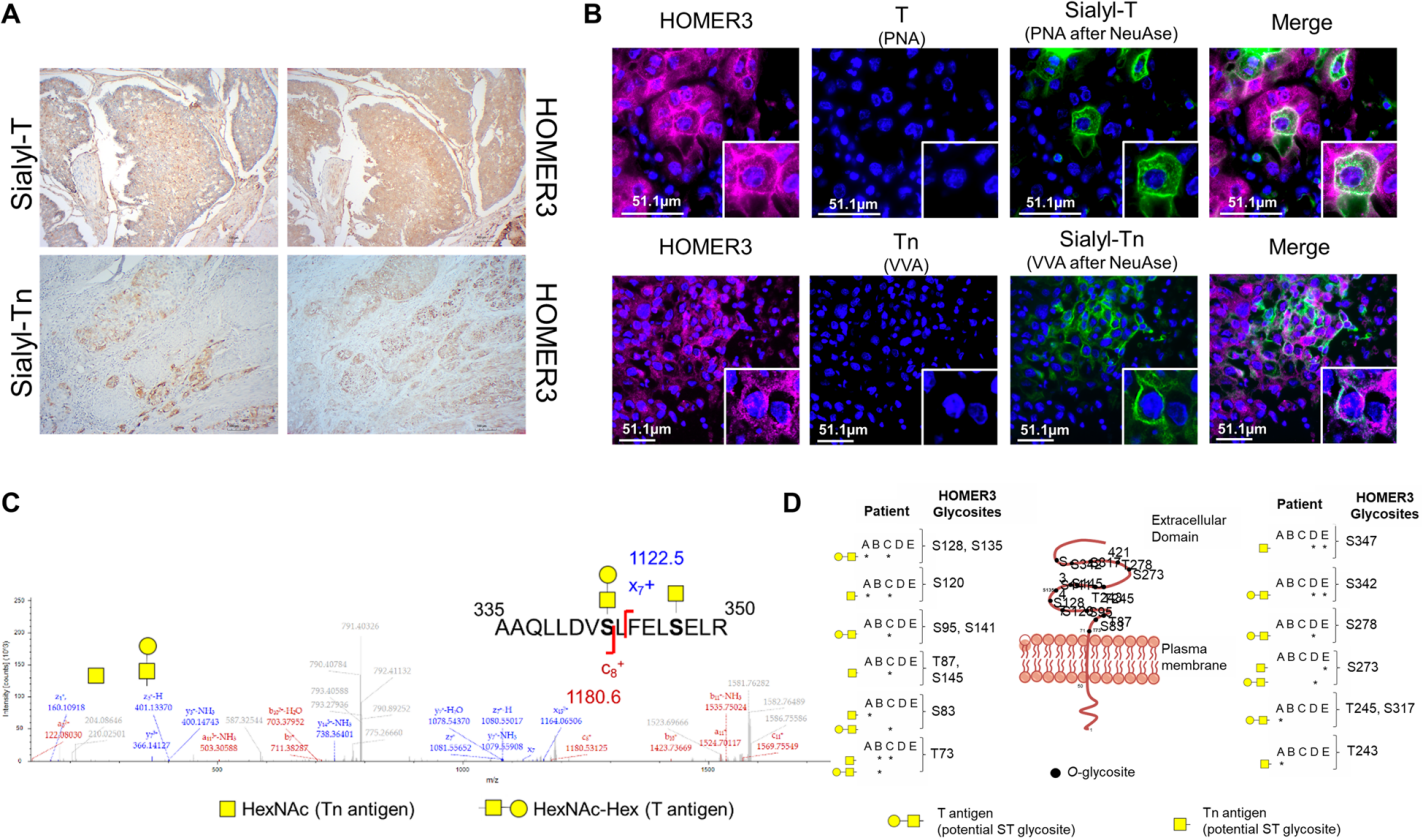
Sialylated HOMER3 glycoforms can be found at the cell-surface of bladder cancer cells and display significant inter-patient structural variability. **A**) HOMER3 at the cell membrane co-localizes in the same tumour area with sialylated Tn and T antigens in bladder cancer. According to immunohistochemistry, HOMER3 was diffusely expressed in wide tumour areas, both at the cytoplasm and cell membranes. These areas co-localized with very high STn and/or ST expressions areas. **B**) Immunofluorescence microscopy highlighting the co-localization (in white) of HOMER3 (violet) with sialylated Tn and T antigens (green) at the cell membrane of bladder cancer cells. HOMER3 was detected in both cytoplasm and cell membrane of a high number of cancer cells in invasive tumours. The tumours were also screened for the Tn and T antigens and their sialylated counterparts STn and ST. Tumours were negative for neutral glycans but showed high sialospecies, in accordance with immunohistochemistry analysis in panel A. **C**) MS/MS HOMER3 glycopeptide with cancer-associated glycans isolated from an invasive bladder tumour. Briefly, invasive tumours showing no Tn and T antigens were elected for this analysis. Glycoproteins were extracted from these tumours and digested with neuraminidase to expose Tn and T antigens derived from their sialylated counterparts. VVA and PNA lectins were then used to pulldown glycoproteins carrying these glycans for downstream analysis by nanoLC-MS/MS. Peptide and glycopeptide fragmentations as well as typical glycan oxonium ions at *m/z* 204.09 (GalNAc) and 366.14 (GalNAc-Gal) were used for glycoproteins and glycosites annotations. **D**) HOMER3 glycosites mapping by nanoLC-EThcD-MS/MS demonstrated significant inter-patients’ variability. HOMER3 was isolated from five tumour samples of different patients by lectin affinity and characterized in relation to their glycosylation pattern by nanoLC-EThcD-MS/MS. This analysis identified 16 glycosites in the extracellular region of HOMER3 with little homology between different patients. Notably, the identification of glycopeptides carrying the Tn and T antigens strongly supports previous sialylation, since the neutral glycans were not detected in the original tumours

Targeting HOMER3 at the cell membrane requires a careful understanding of its glycosylation patterns. Given the high density of *O*-glycosylation sites in HOMER3 and building on glycomics analysis from bladder cancer cell lines and tumours, we have devoted to a comprehensive mapping of HOMER3 glycoforms in tumour tissues with the objective of setting a molecular rationale for targeted interventions. Five MIBC tumours displaying altered glycosylation, translated by the overexpression of both STn and ST antigens, were selected for this study. We started by observing that HOMER3 positive tumour areas co-localized with ST and STn antigens (Figs. 7A and B), suggesting close spatial proximity. Prior to glycoproteomics analysis, the tissues were also screened and found to be negative for the Tn and T antigens, the neutral forms of the STn and ST antigens as well as for blood group A determinants, that may compete for VVA lectin affinity. These observations were key to design appropriate lectin-based glycoprotein enrichment strategies and targeted glycoproteomics. Briefly, to isolate STn-expressing glycoproteins we have used the VVA lectin after digesting the samples with neuraminidase. The absence of blood group A antigens and the Tn antigen that also show affinity for this lectin ensure that enrichment was achieved based on STn groups. A second-dimension affinity chromatography with PNA was then introduced to isolate glycoproteins carrying sialylated T antigens. Isolated glycoproteins were further digested with chymotrypsin and analyzed by nanoLC-EThcD-MS/MS, resulting in the identification of 16 glycosites in amino acid residues in extracellular domains (Figs. 7C and D; Table S5). Glycopeptides potentially presenting STn (exhibiting GalNAc as PTM after neuraminidase digestion) and ST antigens (GalNAc-Gal as PTM) or both PTMs were detected in all samples (Fig. 7D), confirming the glycosylation of HOMER3 at the cell surface, as previously suggested by glycoproteomics and immunofluorescence microscopy (Fig. 3). This preliminary glycoproteomics screening also showed significant inter-patient variability concerning glycosites occupation and glycosylation patterns (Fig. 7D). This suggested that targeted therapeutics for glycosylated HOMER3 may require multi-valent approaches capable of covering a wide number of patients as well as personalization. Nevertheless, more in depth glycoproteomics studies covering a wide number of patients will be required to identify potentially targetable glycodomains.

### HOMER3 and STn in human tissues

**Fig. 8:**
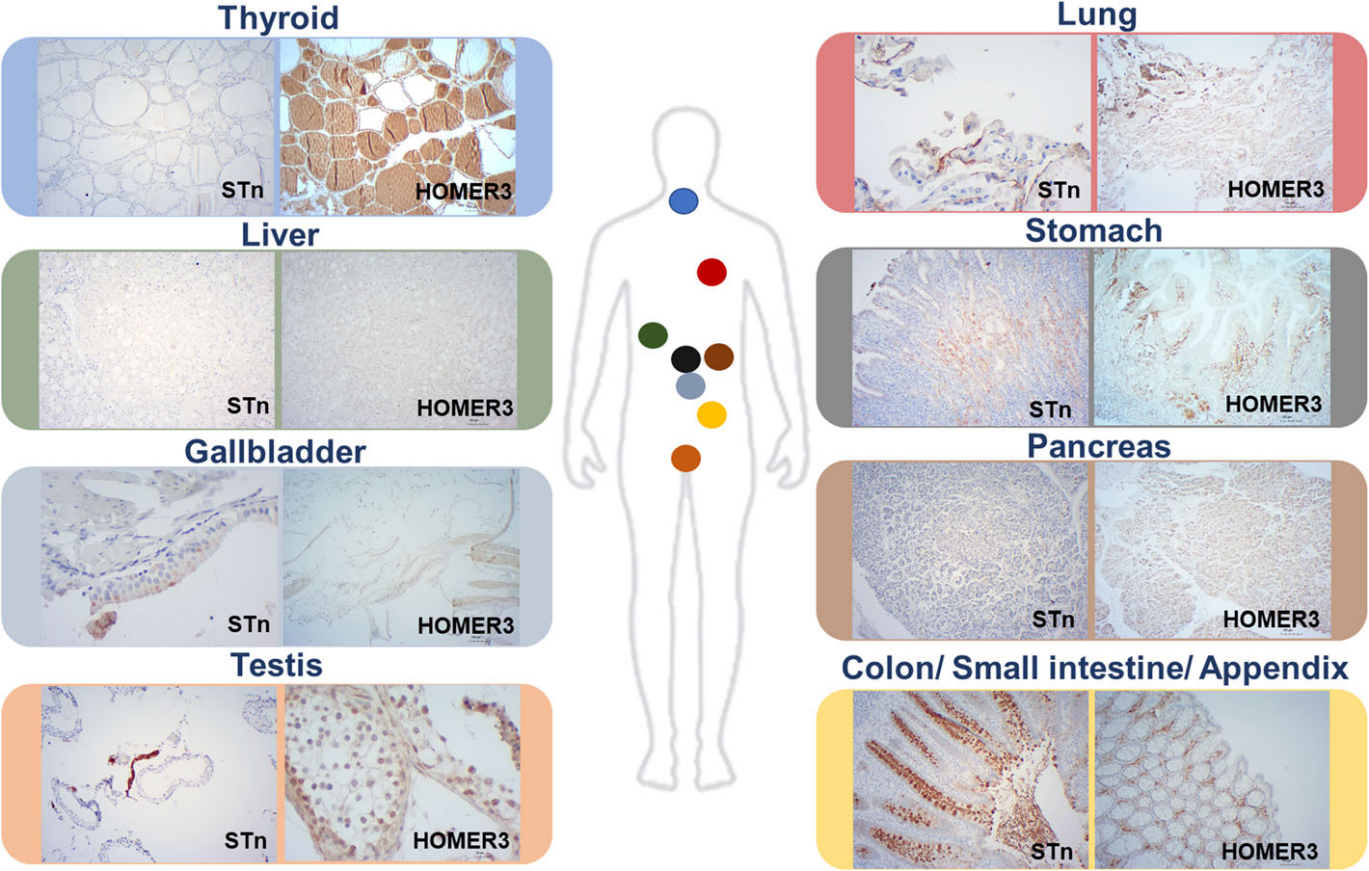
HOMER3 and STn are not co-expressed at the cell membrane in relevant human healthy tissues (thyroid, liver, gallbladder, testis, lung, stomach, pancreas, colon, small intestine, appendix). HOMER3 was not detected in these tissues, except for few cells in the submucosa of digestive organs as well as in thyroid follicular cells, all of which showing weak/moderate cytoplasmic staining. HOMER3 was also detected in respiratory tract secretions, suggesting potential translocation of the protein to the extracellular space, warranting confirmation in future studies. Moreover, HOMER3 was not found at the cell surface of healthy cells, contracting with observations for cancer cells. The STn antigens could only be found in mucinous secretions of the gastrointestinal and respiratory tracts, being also scarcely observed at the cell surface of cells facing the lumen of these organs. No evidence of co-localization between both antigens could be observed

To ensure precise targeting of more aggressive cancer cells and identify potential off-target effects, HOMER3 expression was also evaluated in a subset of relevant healthy tissues (thyroid, liver, gallbladder, testis, lung, stomach, pancreas, colon, small intestine) by immunohistochemistry. HOMER3 was not detected in any of the studied tissues, apart from cells in the submucosa of digestive organs and thyroid follicular cells, which showed weak/moderate cytoplasmic expression (Fig. 8). We also observed HOMER3 staining in secretions of the respiratory tract, suggesting protein translocation to the extracellular space, which should be confirmed in future studies. These findings contrast with the intense membrane expression in cancer cells, raising little off-target concerns. In addition, we have screened raw proteomics datasets deposited in the PRIDE repository (project reference PXD000561) for HOMER3. The search comprehended 20 different types of healthy organs/cells from over 24 individuals, corresponding to a total of 497 samples, which are part of the Human Proteome Project. Briefly, raw MS/MS files were reinvestigated with objective of identifying HOMER3 and possibly *O*-glycosylated domains. Among the included organs are the adrenal gland, colon, rectum, esophagus, kidney, heart, liver, ovary, pancreas, lung, gallbladder, prostate, frontal cortex, spinal cord, and testis. Immune cells such as B cells, CD8^+^ T cells, CD4^+^ T cells, NK cells and monocytes were also studied. HOMER3 was only detected in kidney; however, no glycosites presenting typical cell membrane glycans could be identified, suggesting a cytosolic protein sublocalization. Proteomics results, including the possible presence of HOMER3 in the kidney, should be complemented in the future with more detailed histological analysis for definitive confirmation.

Finally, we have screened the same healthy tissues for the STn antigen, which was the most relevant HOMER3 glycosylation modification across different bladder cancer patients. The STn antigen was not detected in the healthy urothelium (data already published), in accordance with previous studies [12, 18, 22] (Fig. 8). Moreover, it could only be observed in mucin secretions of the gastrointestinal tract but not at the cell membrane of cells (Fig. 8). Immunohistochemistry studies also suggest that STn and HOMER3 are not found together at the cell surface of healthy tissues, reinforcing the cancer-associated nature of the HOMER3-STn glycophenotype. Therefore, this study supports HOMER3-STn as a cancer-specific targetable biomarker to address more aggressive subpopulations of cancer cells in hypoxic niches.

## Discussion

The management of advanced stage bladder cancer patients remains challenging due to the lack of efficient targeted therapeutics, urging the identification of novel and more cancer-specific biomarkers. Envisaging this goal, we have previously demonstrated that cancer cells experience significant glycoproteome remodeling resulting from changes in gene expression, protein processing and maturation, including alterations in glycosylation [6, 18, 22]. Furthermore, we and other authors have highlighted that hypoxia and nutrient deprivation resulting from poor tumour vascularization, constitute relevant microenvironmental features leading to these changes [18–20]. This often originates cancer-specific molecular signatures that drive key oncogenic hallmarks and hold tremendous potential for clinical intervention [5]. Nevertheless, studying the membrane glycoproteome remains challenging, requiring a customization of conventional proteomics protocols to accommodate the structural subtleties originated by glycosylation. Moreover, the glycobiology field has not yet started to explore the full potential of web-available molecular data repositories to comprehensively integrate its findings. This work has addressed these limitations by systematizing a bioinformatics-assisted roadmap for comprehensive interrogation of the cancer glycoproteome for potentially targetable biomarkers. We started by the *O*-glycomics characterization of two relevant bladder cancer cell models showing high invasion capacity, which revealed ST antigens as the most abundant *O*-glycoforms at the cell membrane. This was later confirmed in bladder tumours. Notably, the ST antigens were also detected in healthy urothelium, highlighting lack of tumour specificity. On the other hand, the STn antigen is less expressed by superficial and less aggressive tumours but increases with the severity of the disease. Taken together with our previous reports [12, 18, 22], it is now possible to draw a more detailed picture of the *O*-glycomics landscape associated with bladder tumour progression. It becomes apparent that ST overexpression is part of the initial oncogenic transformation of the bladder, being progressively replaced by the less-extended glycan STn as disease advances. Notably, neither the STn nor the ST antigens present the necessary cancer specificity to support targeted therapeutics, requiring a more comprehensive interrogation of the glycoproteome for biomarker biospecificity. Nevertheless, these insights on the *O*-glycome were key for guiding glycobiomarker discovery and ultimately glycoprotein annotation by mass spectrometry.

Based on these observations, glycan-targeted glycoproteomics in cell lines identified over 900 glycoproteins, many of them showing cell line specificity. This information was comprehensively integrated with available transcriptomics and proteomics data to sort glycoproteins potentially overexpressed in advanced bladder tumours. As a result, the initial list was narrowed down to 95 glycoproteins, including a subgroup associated with bladder cancer aggressiveness, reinforcing the relevance of our analytical approach. Among these glycoproteins were MUC16 and CD44, which have already been extensively studied in the context of bladder cancer as part of more aggressive molecular phenotypes [22]. A *target score* exploring available data on protein expression in tumours and healthy tissues was then developed to rank the glycoproteins according to its potential for targeted therapeutics. Strikingly, the above-mentioned biomarkers were severely penalized by this scoring system due to its lack of tumour specificity. On the other hand, GLUT1 and HOMER3 emerged as top-ranked glycoproteins, mostly due to a very restricted expression pattern in healthy tissues and limited off-target effects potential. GLUT1 plays a key role in the adaptation of bladder cancer cells to microenvironmental challenges, supporting glucose transport for cancer cells high metabolic demands. On the other hand, HOMER3 is a poorly studied protein in cancer, being herein reported for the first time in bladder cancer and, so far, nothing was yet known about its contribution to disease and biomarker potential. As such, focus was set on this protein. Interestingly, HOMER3 is an intracellular protein with a restricted expression pattern in healthy tissues; however, in bladder cancer it was found at the cell surface carrying sialylated short-chain *O*-glycans, which are typically extracellular glycans. From a biomarker standpoint these were crucial observations supporting the trafficking of intracellular proteins to the cell surface concomitantly with the acquisition of aberrant glycosylation traits in cancer cells. According to previous observations, this appears to be a frequent behavior of more aggressive cancer cells [23, 36]. Moreover, these events may significantly contribute to the creation of unforeseen cancer-specific glycosignatures, holding tremendous potential for precise prognosis and targeted therapeutics.

As such, emphasis was first devoted to understanding the microenvironmental context driving HOMER3 accumulation at the cell surface and its contribution to disease. According to our observations, hypoxia and glucose deprivation, two synergic events experienced by cancer cells as result of poor tumour vascularization, drive HOMER3 translocation to the cell membrane. Using gene editing strategies, we have demonstrated that the presence of HOMER3 at the cell membrane significantly enhanced cancer cells proliferation and invasion by yet unknown molecular pathways, which should be identified in future studies. Notably, functional role played by HOMER3 at the cell surface appears dependent on the microenvironment, showing more impact on proliferation in normoxia and contributing to invasion in hypoxia and glucose deprivation. However, these features cannot be easily anticipated without a thorough understanding about the HOMER3 interaction, which will be comprehensively addressed in future studies. Nevertheless, in accordance with its functional role, the membrane HOMER3 phenotype was associated with more advanced stages of the disease and detected in the corresponding metastases, reinforcing its potential for targeted therapeutics capable of controlling disease dissemination. Furthermore, HOMER3 has been identified as an independent predictor of poor prognosis in bladder cancer. Collectively, these findings set a novel biomarker panel for prognosis in bladder cancer and may provide means to target hypoxic niches, that harbor highly aggressive cancer cells. Finally, we have observed that HOMER3 in bladder tumours is glycosylated with simple sialylated *O*-glycoforms, in agreement with observations from cell models. Namely, HOMER3 was found carrying the cancer associated STn antigen, which is rarely observed in healthy organs and significantly overexpressed by more aggressive bladder tumours and, particularly, hypoxic cells [12, 18]. Strikingly, the STn antigen is also an independent predictor of poor prognosis and a promotor of invasion and immune escape in bladder cancer [12, 18, 21]. As such, we postulate that HOMER3-STn glycoforms may be key for precise cancer targeting. In fact, observations arising from a wide array of healthy tissues strongly suggest that HOMER3 localization at the cell membrane is not common and may be characteristic of cancer cells. Moreover, no simultaneous expression of HOMER3 and STn was observed after analysis of over 400 different non-pathological samples from more than 20 different tissues/cell types, setting the molecular foundations for developing novel therapeutics with potentially limited off-target effects.

In summary, despite the preliminary nature of these findings facing clinical translation, our bioinformatics-assisted multi-omics platform has demonstrated potential for generating relevant molecular information for guiding future studies. Moreover, it has identified HOMER3 as top-ranked targetable glycoprotein for targeting invasive cancer cells and metastases. The presence of this glycoprotein at the cell surface was responsible for massive increase in cell invasion in vitro, which now warrants further confirmation in vivo. The identification of the molecular pathways mediated by this protein will also be a key aspect for intervention. Finally, the glycoproteomics characterization of a small set of muscle-invasive bladder tumours showing similar histological and glycosylation features has generated promising results, showing common glycophenotypes holding potential for precision oncology. A comprehensive mapping of HOMER3 glycosites for cancer neoantigens in a broader and more diversified array of tumour samples and healthy tissues is required, foreseeing the establishment of highly effective therapeutics.

### Concluding remarks

The bladder cancer glycoproteome may be unveiled by targeted glycoproteomics supported by pre-existing multi-omics data, showing tremendous potential for targeted therapeutics. The materialization of this objective has led to HOMER3 identification, a generally intracellular protein presenting a restricted expression pattern in human healthy tissues. We concluded that more aggressive bladder tumours and its metastases exuberantly expressed HOMER3 at the cell surface modified with sialylated short-chain *O*-glycans typical of membrane proteins. According to our study, the HOMER3 cell surface phenotype is an independent predictor of prognosis in bladder cancer triggered by hypoxia and glucose deprivation. It also plays a major role enhancing cell proliferation and invasion, depending on environmental cues. Moreover, this study supports the cancer specific nature of HOMER3-STn glycoforms, setting the rationale for precision oncology, including the development of targeted therapies.

## Supplementary Information

**Additional file 1.**

**Additional file 2.**

## Data Availability

Not applicable.

## Abbreviations

BC: Bladder cancer
BrdU: Bromodeoxyuridine
CA125: Cancer antigen 125
*Cas9*: CRISPR associated protein 9
CID: Collision-induced dissociation
CRISPR: Clustered regularly interspaced short palindromic repeats
ESI: Electrospray ionization
EThcD: Electron-transfer/higher-energy collision dissociation
FFPE: Formalin-fixed paraffin-embedded
gRNA: Guide ribonucleic acid
HOMER3: Homer protein homolog 3
KD: Knock-down
KI: Knock-in
KO: Knock-out
LC: Liquid chromatography
MIBC: Muscle invasive bladder cancer
MS: Mass spectrometry
MUC16: Mucin 16
NMIBC: Non-muscle-invasive bladder cancer
OS: Overall survival
PI: Propidium iodide
PNA: Peanut agglutinin
PSM: Peptide-spectrum match
SLC2A1/GLUT1: Solute carrier family 2, facilitated glucose transporter member 1 / glucose transporter type 1
ST: Mono- and di-sialyl T antigens
ST3GAL1: Beta-galactoside alpha-2,3-sialyltransferase 1
STn: Sialyl-Tn
VVA: *Vicia villosa* agglutinin
