# Supplementary material for: Glycoproteomics identifies HOMER3 as a potentially targetable biomarker triggered by hypoxia and glucose deprivation in bladder cancer
Source: J Exp Clin Cancer Res. 2021 Jun 9;40:191. doi: 10.1186/s13046-021-01988-6 (PMC8188679; doi:10.1186/s13046-021-01988-6)
Supplement: Supplementary file 2 — Additional file 2. [file 13046_2021_1988_MOESM2_ESM.docx]

**Glycoproteomics identifies HOMER3 as a targetable biomarker triggered by hypoxia and glucose deprivation in bladder cancer**

Andreia Peixoto^1,2,3,4^, Dylan Ferreira^1,3,4^, Rita Azevedo^1^, Rui Freitas^1^, Elisabete Fernandes^1,3,4^, Marta Relvas-Santos^1,2,3,4,5^, Cristiana Gaiteiro^1,2^, Janine Soares^1,2^, Sofia Cotton^1,2^, Beatriz Teixeira^1^, Paula Paulo^6^, Luís Lima^1^, Carlos Palmeira^1,7,8^, Maria José Oliveira^3,4^, André M. N. Silva^5^, Lúcio Lara Santos^1,2,8,9,10^, José Alexandre Ferreira^1,2,10^

^1^Experimental Pathology and Therapeutics Group, Research Center (CI-IPOP), Portuguese Institute of Oncology, 4200-072 Porto, Portugal; ^2^Institute of Biomedical Sciences Abel Salazar (ICBAS), University of Porto, 4050-313 Porto, Portugal; ^3^Institute for Research and Innovation in Health (i3S), University of Porto, 4200-135 Porto, Portugal; ^4^Institute for Biomedical Engineering (INEB), University of Porto, 4200-135 Porto, Portugal; ^5^REQUIMTE-LAQV, Department of Chemistry and Biochemistry, Faculty of Sciences of the University of Porto, 4169-007 Porto, Portugal; ^6^Cancer Genetics Group, Research Center (CI-IPOP), Portuguese Oncology Institute of Porto (IPO Porto), 4200-072 Porto, Portugal; ^7^Immunology Department, Portuguese Institute of Oncology of Porto, 4200-072 Porto, Portugal; ^8^Health School of University Fernando Pessoa, 4249-004 Porto, Portugal; ^9^Department of Surgical Oncology, Portuguese Institute of Oncology, 4200-072 Porto, Portugal; ^10^Porto Comprehensive Cancer Center (P.ccc), 4200-072 Porto, Portugal

**Corresponding author:**

José Alexandre Ferreira (jose.a.ferreira@ipoporto.min-saude.pt)

Experimental Pathology and Therapeutics Group, Research Center of Portuguese Oncology Institute of Porto, R. Dr. António Bernardino de Almeida 62, 4200-072 Porto, Portugal; Tel. +351 225084000 (ext. 5111).

**Running head:** HOMER3 as a targetable glycoprotein in bladder cancer


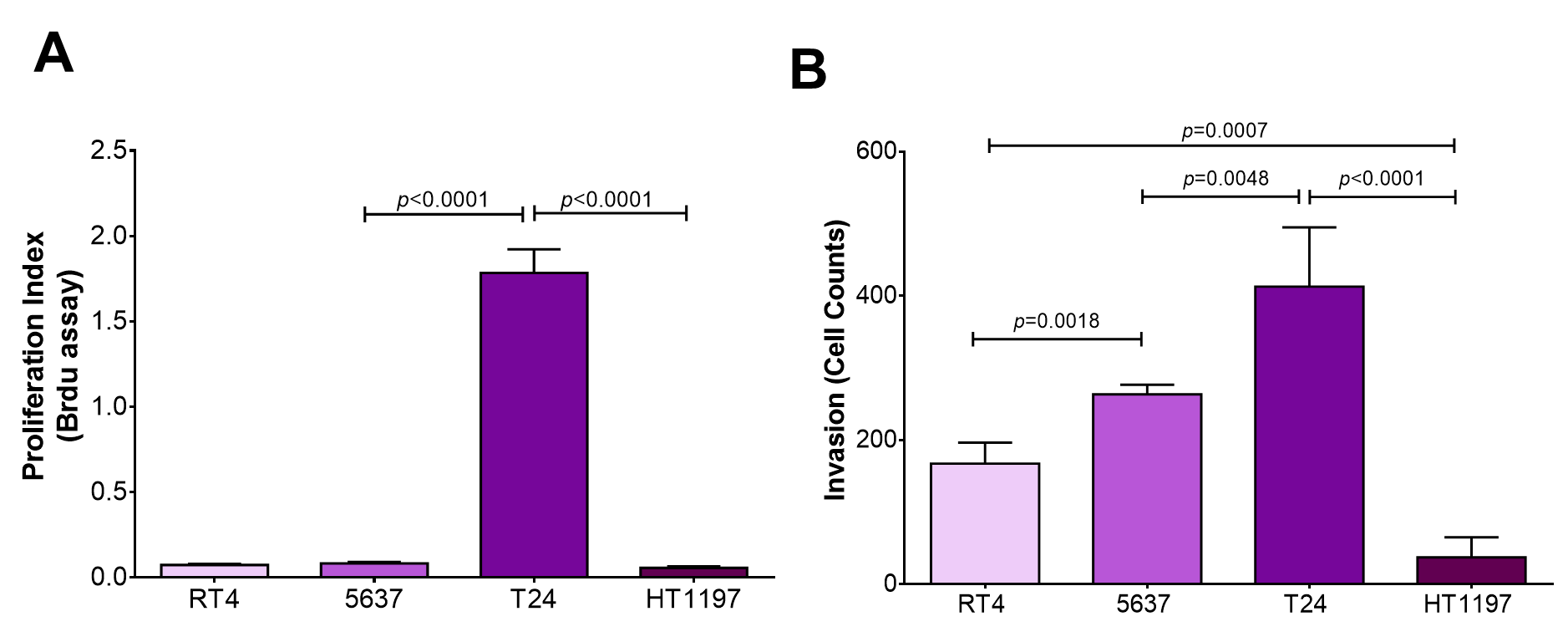


**Figure S1. Estimation of A) cell proliferation and B) invasion of Matrigel for distinct bladder cancer cell models supports the increased aggressiveness of 5637 and T24 cell lines.** Graph A shows that T24 is significantly more proliferative than the other cell lines. Graph B shows that T24 and 5637 are the most invasive cells. Results are the average of three independent replicates. (student t-test).

**
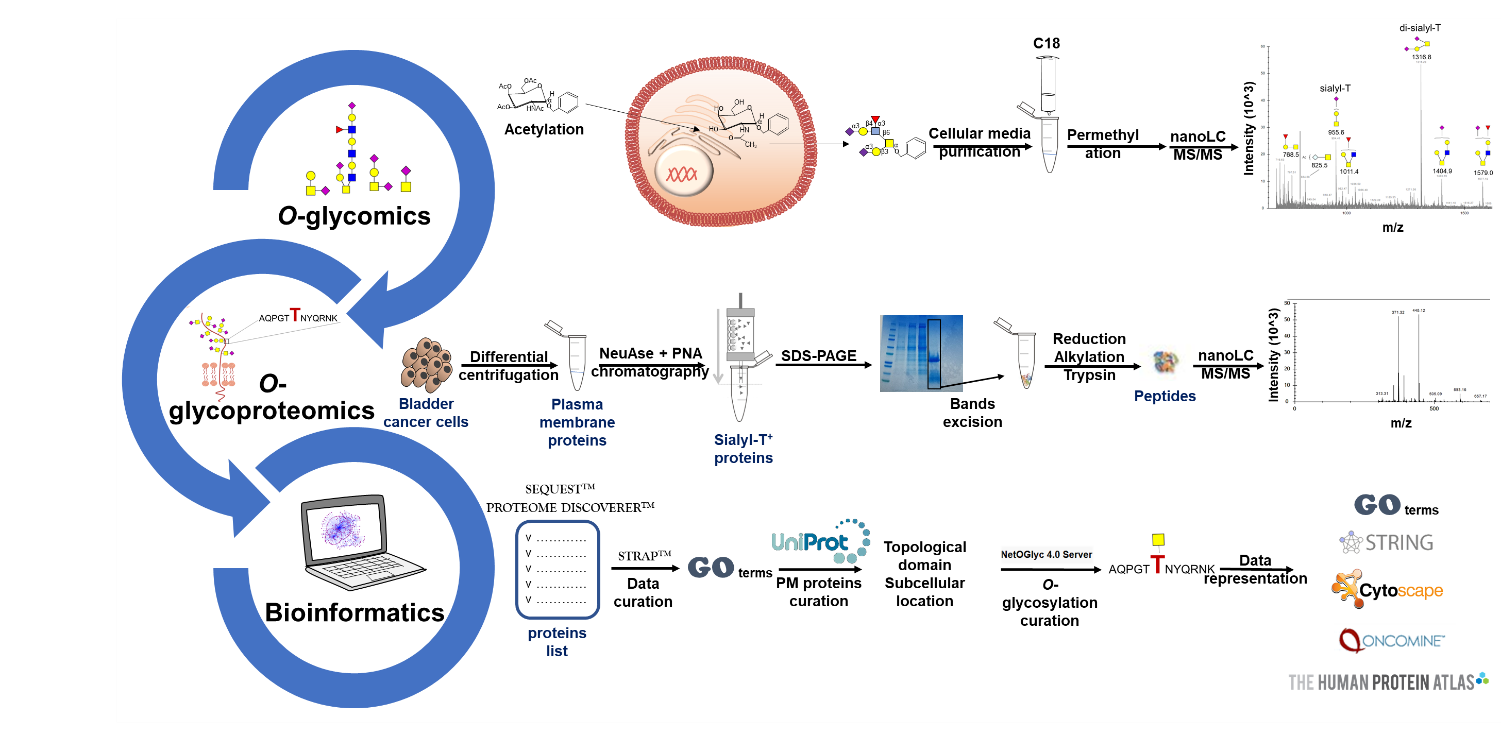
**

**Figure S2. Schematic illustration for the glycoproteins identification workflow.** The high glycosylation frequency and glycosites density presented by membrane glycoproteins poses a significant analytical hurdle to conventional proteomics protocols. To address this limitation, we have established a three-step approach, combining *O*-glycomics, *O*-glycoproteomics and bioinformatics. *O*-glycomics characterization was performed through the Cellular *O*-glycome Reporter/Amplification method, which exploits a glycan mimetic of the simplest form of *O*-glycosylation, serving as scaffold for endogenous glycosylation extension. Briefly, this glycan mimetic is first peracetylated to render it more hydrophobic to passively diffuse through the plasma membrane. Inside the cell, the glycan precursor is rapidly deacetylated by intracellular deacetylases, glycosylated by the available glycosylation machinery and secreted back into the extracellular medium. After recovery with C18 reverse phase cartridges, glycans were permethylated to make them more hydrophobic, facilitate ionization in positive mode and stabilize labile sugars, followed by C18 nanoLC-MS/MS analysis. Glycome characterization allowed designing an *O*-glycoproteomics protocol based on an enrichment strategy by peanut agglutinin (PNA) lectin affinity chromatography for identification of sialylated glycoproteins. Briefly, samples were digested with neuraminidase exposing cryptic T antigens by sialylation, increasing glycoproteins affinity for PNA. After enrichment glycoproteins were reduced, alkylated, digested with trypsin, and subsequently identified by bottom-up nanoLC-CID-MS/MS-based proteomics. Data was curated for glycoproteins of interest based on different bioinformatics strategies. The initial list of protein identifications was first curated for proteins with probability to exist at the cell surface by gene ontology (GO terms) and then evaluated for the probability to be *O*-glycosylated using the NetOGlyc 4.0 server (<http://www.cbs.dtu.dk/services/NetOGlyc/>). The final glycoproteins list was then manually screened for peptides containing glycans. The final data was comprehensively interpreted by cross-reference with cancer databases (Oncomine, Human Protein Atlas) to sort potentially targetable glycobiomarkers.

**
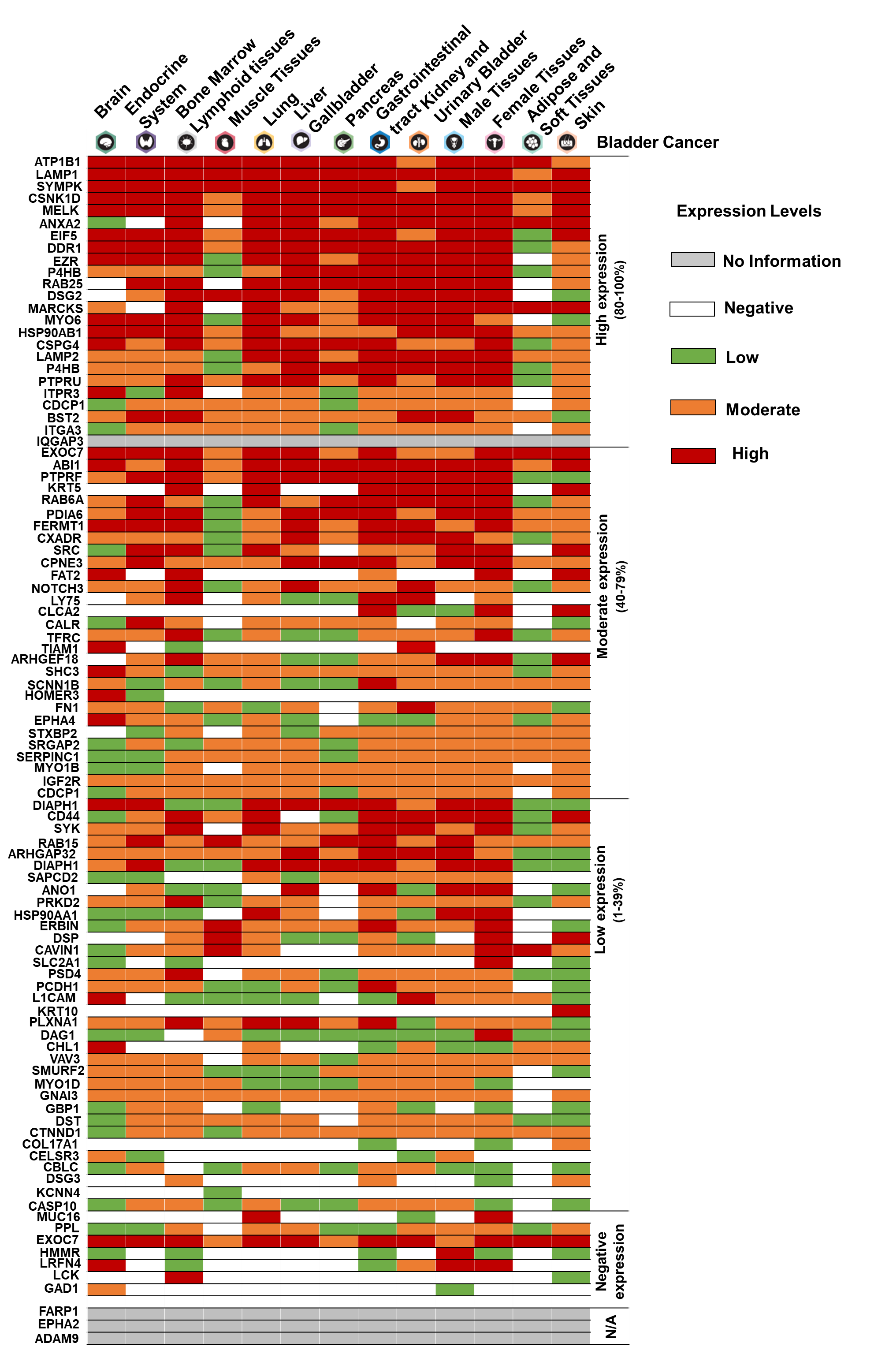
**

**Figure S3. Levels of expression of glycoproteins identified by targeted glycoproteomics in different human tissues and bladder cancer.** The glycoproteins identified in 5637 and T24 cells were categorized according to the expression in bladder cancer and a wide array of human tissues, according to The Human Protein Atlas. This matrix was used to support the estimation of the *target score*.


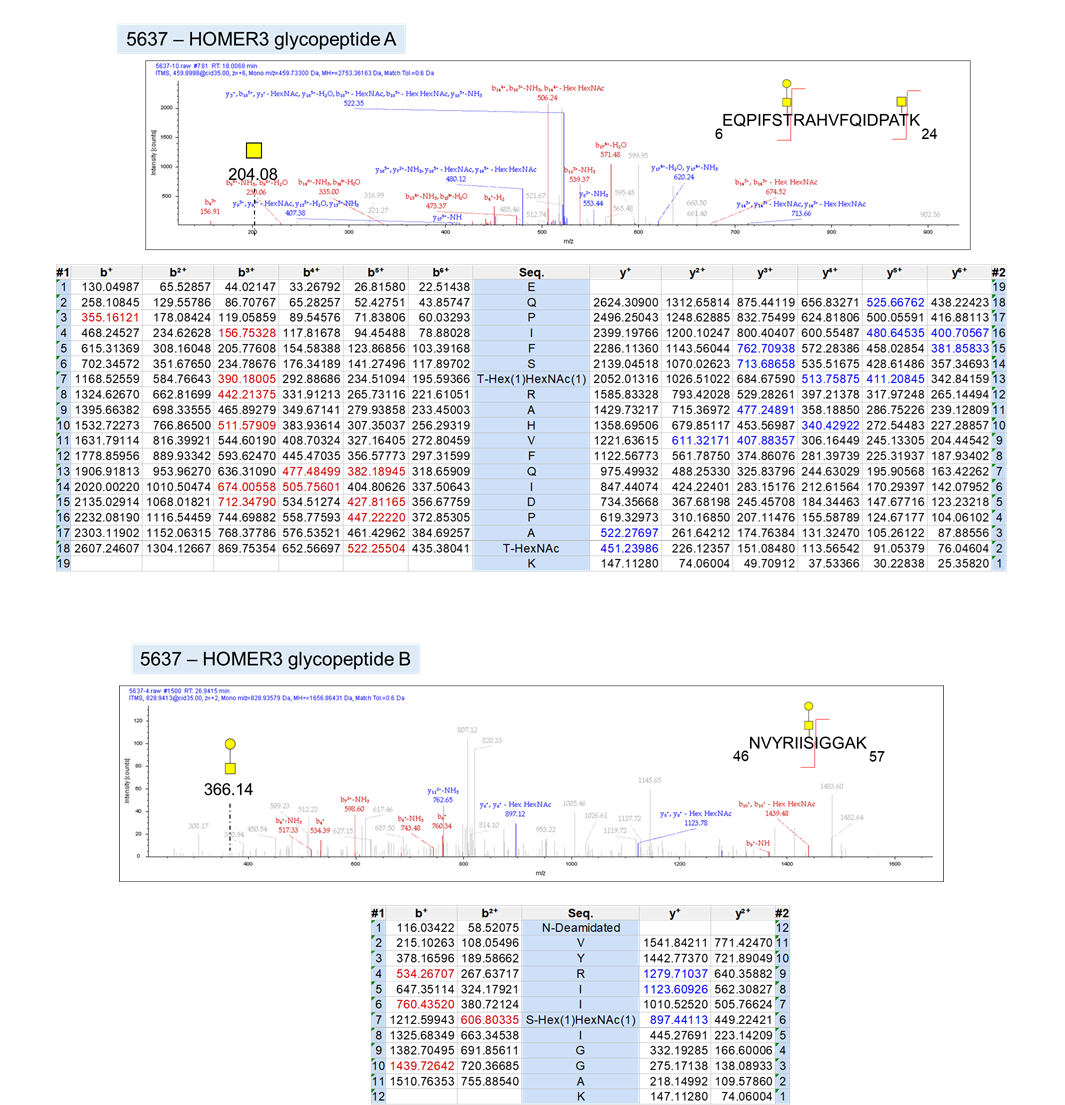


**Figure S4. Examples of MS/MS spectra for HOMER3 glycopeptides identified in cell line 5637.** Annotated product ion spectra for two different HOMER glycopeptides identified in cell line 5367. The spectra show glycan oxonium ions at *m/z* 204.08 and/or 366.14, characteristic of HexNAc and Hex-HexNAc. Also, b- and y-series peptide fragments allowed precise glycosite annotation for these glycopeptides. Collectively, these two glycopeptides confirm the existence of HOMER3 glycoforms in cell line 5637.

**
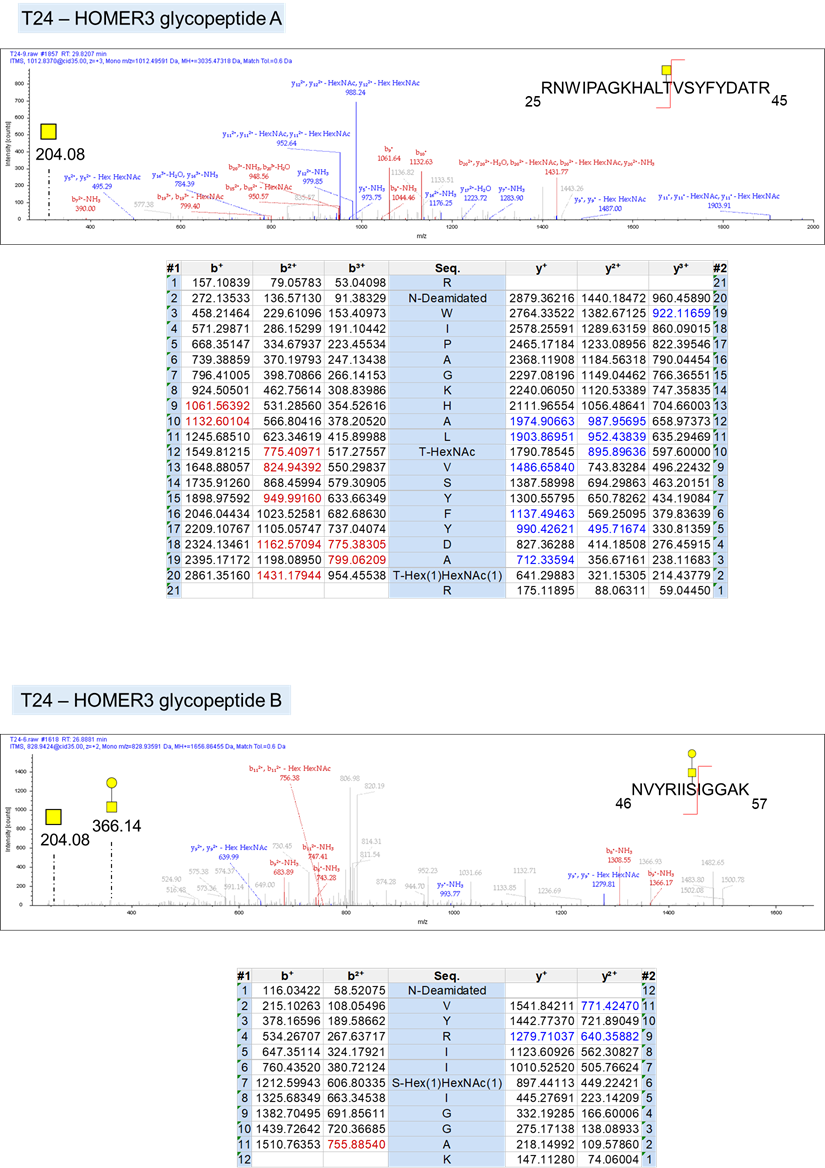
**

**Figure S5. Examples of MS/MS spectra for HOMER3 glycopeptides identified in cell line T24.** Annotated product ion spectra for two different HOMER glycopeptides identified in cell line T24. The spectra show glycan oxonium ions at *m/z* 204.08 and/or 366.14, characteristic of HexNAc and Hex-HexNAc. Also, b- and y-series peptide fragments allowed precise glycosite annotation for these glycopeptides. Interestingly, glycopeptide B was also identified in cell line 5637. Collectively, these two glycopeptides confirm the existence of HOMER3 glycoforms in cell line T24.

**
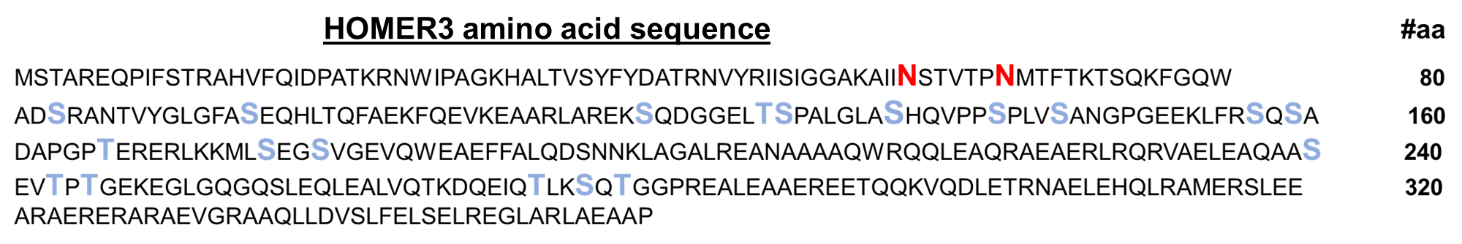
**

**Figure S6. HOMER3 canonical amino acids sequence highlighting a higher density of potential *O*-glycosylation (light blue) in comparison to *N*-glycosylation sites (in red).** Potential *O*-glycosylation sites were determined with using the NetOGlyc 4.0 server (<http://www.cbs.dtu.dk/services/NetOGlyc/>) and *N*-glycosylation was determined by NetNGlyc 1.0 (<http://www.cbs.dtu.dk/services/NetOGlyc/>).

**
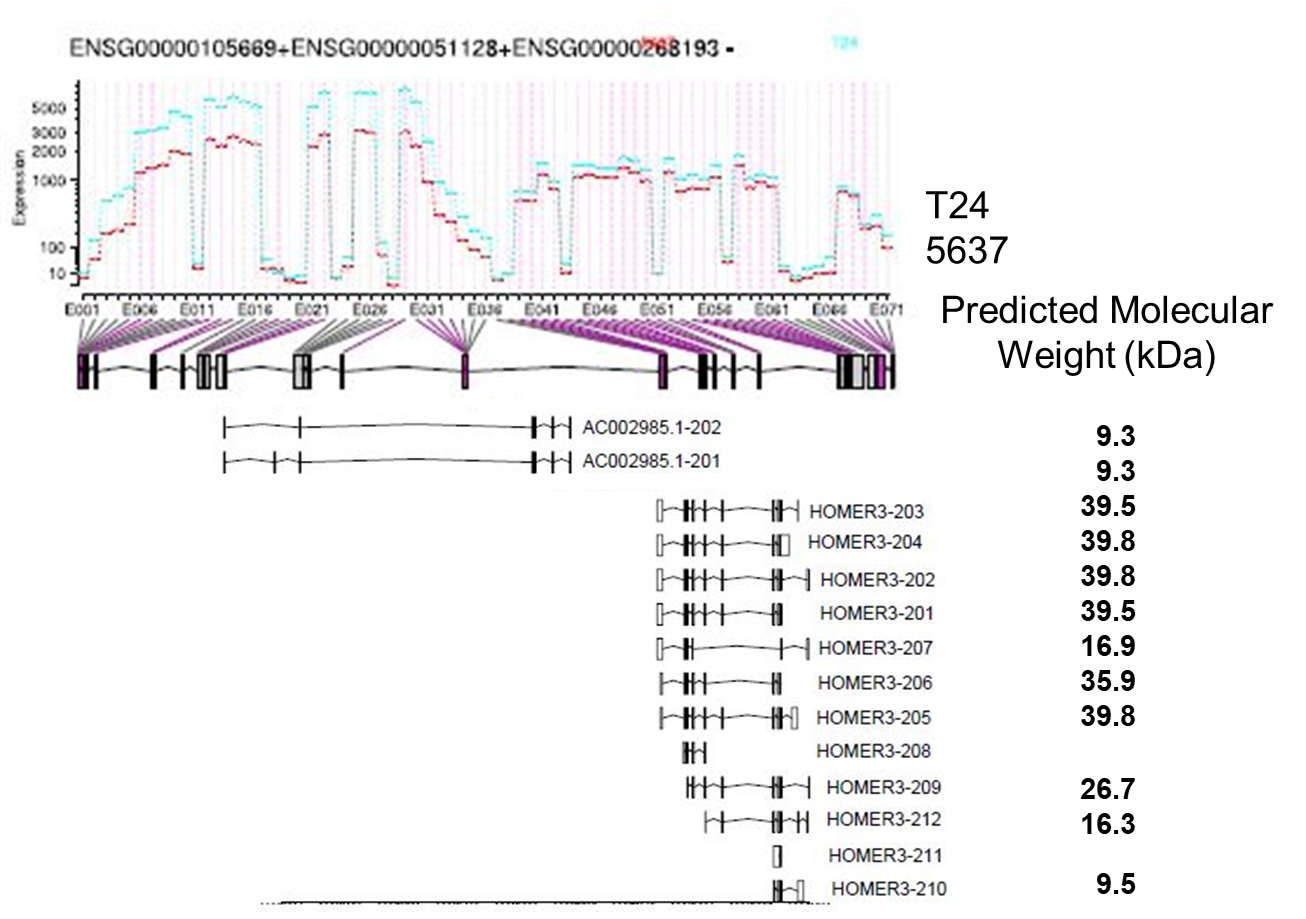
**

**Figure S7. RNAseq for HOMER3 suggesting the existence of multiple proteoforms spanning several molecular weights (10-40 kDa).**

**
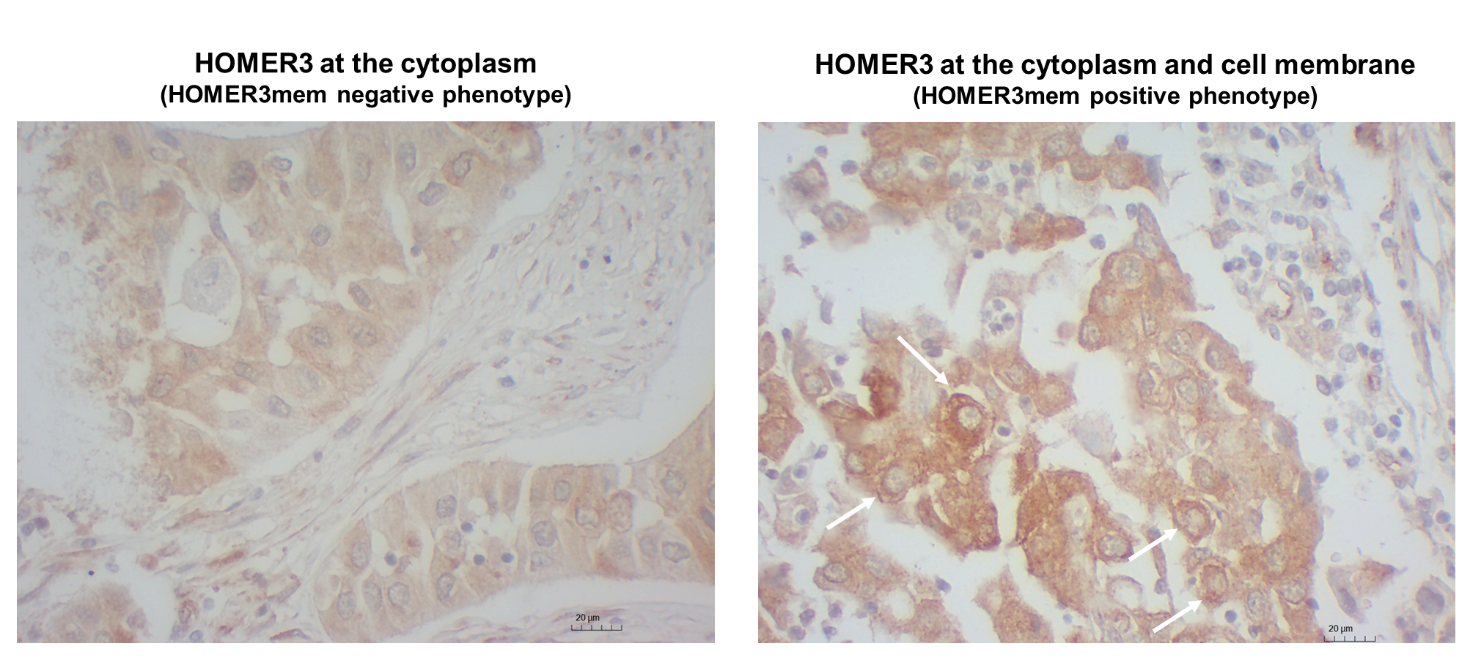
**

**Figure S8. Bladder tumours presenting the HOMER3mem negative (left panel) and HOMER3mem positive (right panel) phenotypes.** Bladder tumours presenting a marked cytoplasm HOMER3 staining and no evident membrane stanning (left panel) were classified as HOMER3mem negative tumours, whereas tumours evidencing cells with clear membrane expression (right panel; highlighted by white arrows) were classified as HOMER3mem positive.

**
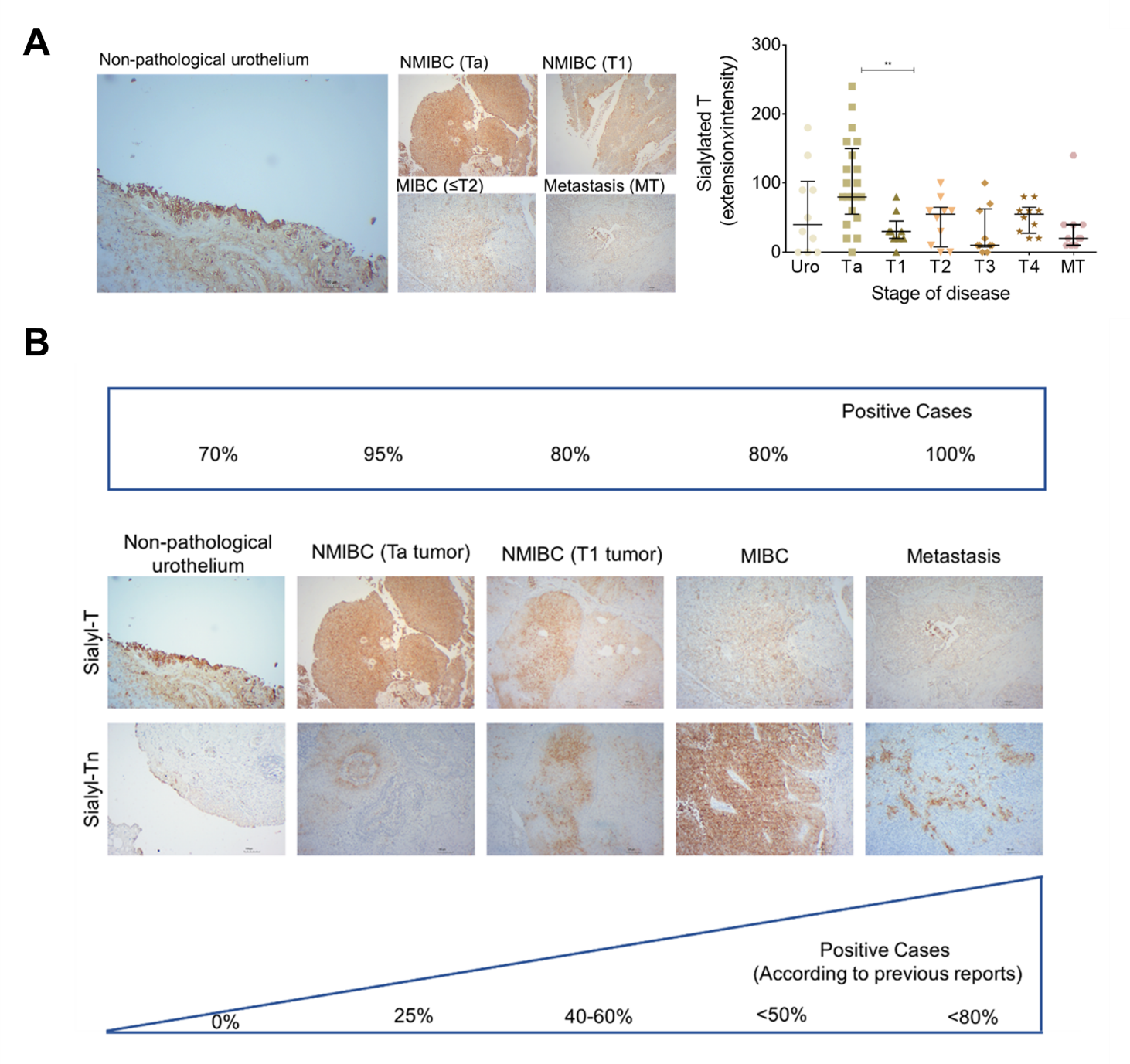
**

**Figure S9. Sialylated T antigens are expressed in the healthy urothelium and increased in cancer, being more pronounced in Ta tumours, in clear contrast with STn antigens, which are characteristic of more aggressive forms of the disease. A) Expression of sialylated T antigens in the healthy urothelium and at different stages of the disease (Ta, T1-4).** Tissues were first screened for the T antigen with PNA lectin, which showed negative to neglectable positivity in all cases. The tissues were then digested with neuraminidase rendering T antigens from sialylated T antigens and reanalysed with the same lectin. ST antigens expression was scored based on intensity *vs* extension of staining. ST antigens were detected on most samples (healthy urothelium, primary lesions, metastases) with high intensity and extension, being more exuberant in Ta tumours. **B)** **Illustrative panel of ST *vs* STn positive cases according to the severity of the disease.** The data presented here concerning STn antigen has been previously published by us (Ferreira et al. Mol Oncol. 2013; Santos, Fernandes and Ferreira et al. PLoS Negl Trop Dis. 2014; Lima et al. Urol Oncol. 2017). The ST antigen is present in the majority of healthy urothelium (70%), but the number of positive cases increases in cancer, with emphasis on the Ta tumours. On the other hand, the STn antigen is not expressed by the healthy urothelium, is expressed by few Ta tumours (approximately 25%) and the number of positive cases more than doubles with the severity of the lesions.
